# Phenotypic Innovation and Adaptive Constraints in the Evolutionary Radiation of Palaeozoic Crinoids

**DOI:** 10.1038/s41598-017-13979-9

**Published:** 2017-10-23

**Authors:** David F. Wright

**Affiliations:** Department of Paleobiology, National Museum of Natural History, Smithsonian Institution, Washington, D.C. 20013 USA

## Abstract

To better understand the patterns and processes shaping large-scale phenotypic diversification, I integrate palaeobiological and phylogenetic perspectives to investigate a ~200-million-year radiation using a global sample of Palaeozoic crinoid echinoderms. Results indicate the early history of crinoid diversification is characterized by early burst dynamics with decelerating morphologic rates. However, in contrast with expectation for a single “early burst” model, morphospace continued to expand following a slowdown in rates. In addition, I find evidence for an isolated peak in morphologic rates occurring late in the clade’s history. This episode of elevated rates is not associated with increased disparity, morphologic novelty, or the radiation of a single subclade. Instead, this episode of elevated rates involved multiple subclade radiations driven by environmental change toward a pre-existing adaptive optimum. The decoupling of morphologic disparity with rates of change suggests phenotypic rates are primarily shaped by ecologic factors rather than the origination of morphologic novelty alone. These results suggest phenotypic diversification is far more complex than models commonly assumed in comparative biology. Furthermore, palaeontological disparity patterns are not a reliable proxy for rates after an initial diversifying phase. These issues highlight the need for continued synthesis between fossil and phylogenetic approaches to macroevolution.

## Introduction

It is often stated that the major features of Earth’s biodiversity are the result of successive evolutionary radiations spanning the geologic history of life^1,2^. Adaptive radiation, a process whereby a clade increases in phenotypic disparity as a result of ecologic opportunity, is the most commonly invoked model of clade diversification and is backed by a rich theory and a wealth of empirical case studies^1–4^. Rates of phenotypic evolution are expected to be maximal during the early stages of adaptive radiation, such as when a lineage enters a new adaptive zone, but declines with increasing clade age as niche space approaches saturation^1,2^. A corollary of this prediction is that morphologic disparity should peak early in a clade’s history, and disparity profiles compatible with “early burst”^5^ diversifications are frequently observed in the fossil record^6–9^. However, similar disparity profiles may arise for reasons not related to ecologic change but because developmental constraints or other factors influence patterns of phenotypic diversification^3,10–13^. In contrast to palaeobiologic studies, phylogenetic comparative analyses of extant species rarely support the early burst model and instead suggest models of constrained diversification around an adaptive peak or Brownian motion-like diffusion through morphospace^5^. Although the lack of support for adaptive radiation in comparative data may reflect statistical difficulties in testing for early bursts^14^, the discordance between fossil and phylogenetic approaches highlights the need to consider alternative models of morphologic diversification and the importance of considering taxonomic and temporal scales when relating pattern to process^9,15^. The view taken here is that palaeontological disparity and phylogeny-based approaches offer complementary ways to examine morphologic evolution, but emphasize different aspects of phenotypic diversification.

To test whether large-scale patterns of morphologic evolution within a long-lived clade are primarily shaped by early burst-like dynamics or increased phenotypic constraint, I apply recently developed methods integrating both phylogenetic and palaeobiologic perspectives to empirically document ~200 million years of morphologic diversification using a major clade of marine invertebrates, the eucladid Crinoidea (sea lilies and feather stars). Eucladids are the most ecologically diverse, species-rich clade of crinoid echinoderms^16–18^ and have a densely sampled Palaeozoic fossil record ideal for studying morphologic change over geologic timescales^6,19^. I compiled a novel dataset comprising 92 discrete morphologic traits spanning the entire crinoid body plan using a global sample of species from 92% of Palaeozoic eucladid families (Supplementary Information) and conducted phylogeny-based likelihood and Bayesian relaxed clock analyses to estimate rates of morphologic evolution (Materials and Methods). To account for uncertainty in tree topology, divergence times, and branch lengths, I estimated mean and median rates of character change over a random sample of time-calibrated trees obtained from a Bayesian posterior distribution of fossil tip-dated phylogenies (Materials and Methods).

## Results and Discussion

**Figure 1:**
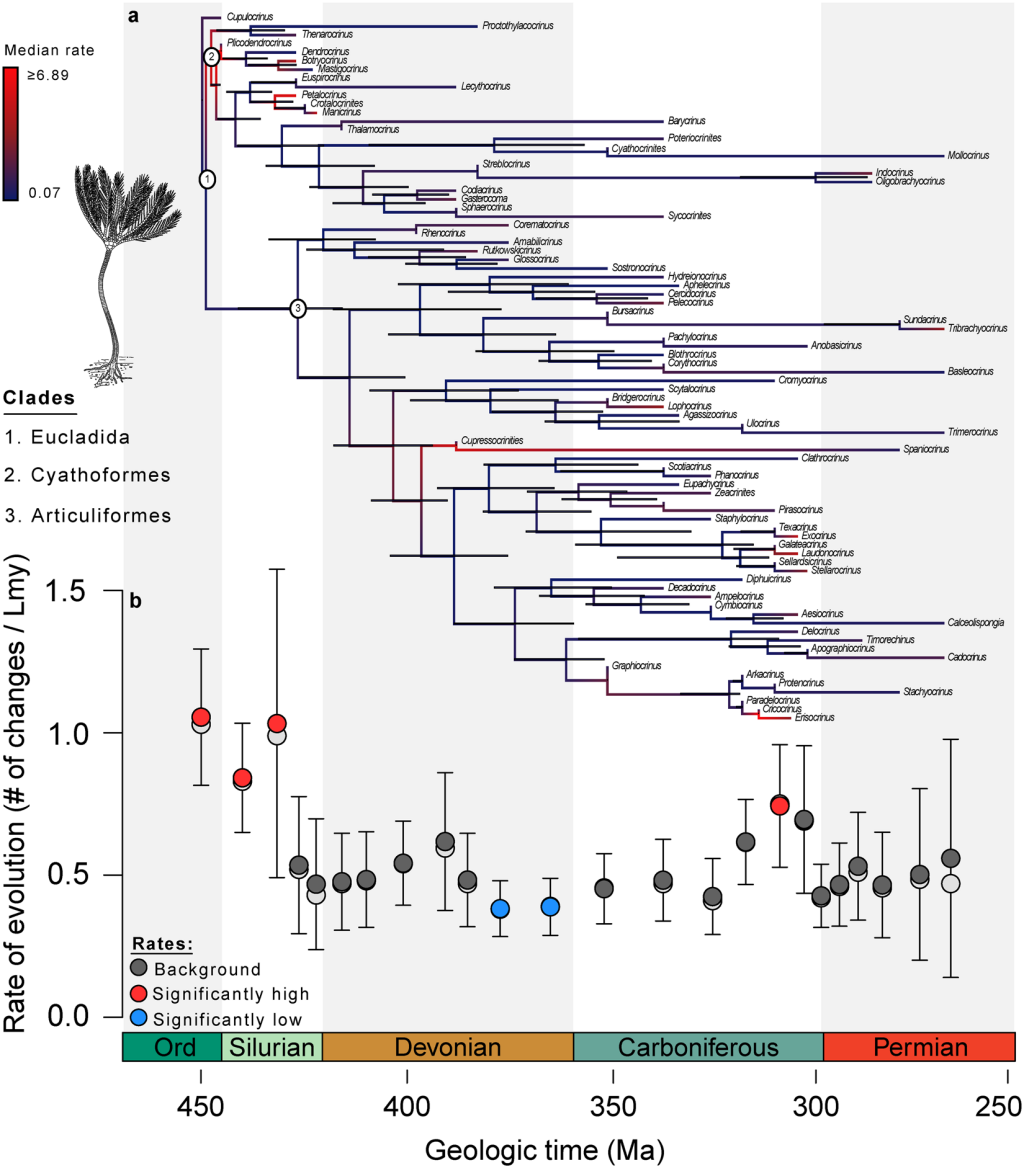
Rates of phenotypic evolution among Palaeozoic eucladid crinoids. (**a**) Maximum Clade Credibility tree from the Bayesian phylogenetic analysis. Major clades are identified at nodes. Median rates of morphologic evolution are shown along branches. Red branches indicate elevated rates and blue branches indicate lower rates. Node bars represent the 95% highest probability densities for divergence dates. (**b**) Results from maximum-likelihood analysis of a random sample of 100 time-calibrated trees from the Bayesian posterior distribution. Colored circles indicate mean rates and open circles represent medians. Intervals with red circles are characterized by statistically high rates using likelihood-ratio tests, whereas intervals with blue circles have statistically low rates (Materials and Methods). Intervals with dark grey circles have rates statistically indistinguishable from the background rate. Error bars are 95% confidence intervals. The abscissa represents time in millions of years (Ma). Reconstruction of the eucladid *Dictenocrinus* from ref.^65^.

Major fluctuations in the rate of morphologic evolution are evident across the Palaeozoic radiation of eucladid crinoids (Fig. 1). Morphologic rates were at their highest early in the Palaeozoic and exhibit a long-term secular decrease in mean rates through time (Spearman’s *Rho* = −0.46, *P* = 0.022). The Late Ordovician to middle Silurian is characterized by elevated rates of character change that are approximately twice as high as most subsequent intervals. Rates significantly drop during the Devonian, reaching their Palaeozoic minimum during the late Devonian Frasnian-Famennian stages. Although mean rates increased during the early Carboniferous, they do not rise above background levels and are comparable to those of the late Silurian to Early Devonian. Rates were elevated throughout much of the late Carboniferous, with a singular burst of morphologic evolution occurring during the Moscovian stage that resulted in the only significant post-Silurian peak in morphologic rates (Fig. 1, Methods and Materials). Although the early to middle Palaeozoic radiation of eucladids is consistent with an early burst pattern, the episodic peak during the late Carboniferous suggests that morphologic diversification during the late Palaeozoic was driven by qualitatively different evolutionary dynamics (Supplementary Information, Fig. S1).

It is important to rule out possible biases that may influence these patterns because variation in rate estimates can arise even when underlying rates of evolution are constant. For example, elevated rates of evolution may be inferred simply because taxa were more densely sampled and therefore more changes can be recorded. A sampling curve of phylogenetic lineages (Supplementary Information, Fig. S2) shows a major peak during the early Carboniferous, which is not associated with high rates of character change. Similarly, sampled lineage diversity was much lower during the Ordovician to middle Silurian than most of the Palaeozoic (Supplementary Information, Fig. S2). A comparison of lineages sampled to the number of all known valid eucladid genera reveals late Carboniferous crinoids are proportionally undersampled in the dataset (taxa and references listed in ref.^16^, Supplementary Information). Thus, the late Carboniferous peak in mean rates would be expected to be even higher if species had been sampled in proportion to taxonomic richness. Sampling biases arising from incompleteness of the fossil record could also affect rate estimates. Palaeontologic metrics assuming uniform preservation^19^ indicate sampling probabilities for Palaeozoic eucladids are slightly higher than estimates for all Ordovician to Devonian crinoid genera (per-interval preservation probability = 0.60, sampling rate = 0.12, see ref.^19^), and suggests sampling is ~92% complete at the scale of analysis presented herein.

Although crinoids as a whole exhibit variation in post-mortem disarticulation rates^20^, studies of taphonomic degradation find similar patterns of disarticulation within major clades such as the Eucladida^20,21^. Thus, there is little *a priori* reason to suspect biased variation in preservation potential among taxa analysed. However, non-uniform preservation of the rock record itself may also influence palaeontologic patterns, especially over geologically long timescales (10^7^–10^8^ years). A stochastic model of non-uniform sampling was incorporated directly into the phylogenetic analysis to account for time-varying rates of fossil sampling (Materials and Methods). Although sampling fluctuated through time (Supporting Information, Fig. S3), mean rates of character change are not significantly correlated with either phylogeny-based estimates of sampling rates (Spearman’s *Rho* = 0.155, *P* = 0.459) or the number of eucladid collections per-bin in the Paleobiology Database (Spearman’s *Rho* = −0.229, *P* = 0.362) (Supporting Information, Figs S3–S6). In summary, the inferred rates of morphologic evolution are robust to a number of potential biases that may affect rate inferences, including variation in tree topology, branch lengths, divergences times, and incomplete or non-uniform fossil sampling.

Disparity studies using similar taxonomic and temporal scales have found that bursts of morphologic evolution occurring after a clade’s early history are commonly associated with major ecologic shifts and rapid diversification of one or more subclades^9,22,23^. Feeding ecology is the primary control on niche differentiation among crinoids^24,25^. Crinoids are passive suspension feeders that depend on current-driven nutrients to feed and have evolved a variety of ecomorphologic traits to help capture food particles and reduce competition among species^26^. Two major eucladid subclades, the Cyathoformes and Articuliformes (Supplementary Information), feature markedly different ecologic and life history strategies. Although cyathoform and articuliform species often co-occur in the same palaeocommunties, their body plan organization reflects adaptations to different ecologic and environmental conditions^26^. Articuliform crinoids have terminal arm appendages called pinnules that serve to increase filtration fan density, which allows for feeding to take place at higher current velocities^27^. Articuliforms also have muscular appendages in the arms that increase motility and enable shifts in feeding posture^28,29^. In contrast, cyathoform crinoids almost universally lack pinnules and have only ligamentary articulations with limited motility^29^ (Supplementary Information).

**Figure 2:**
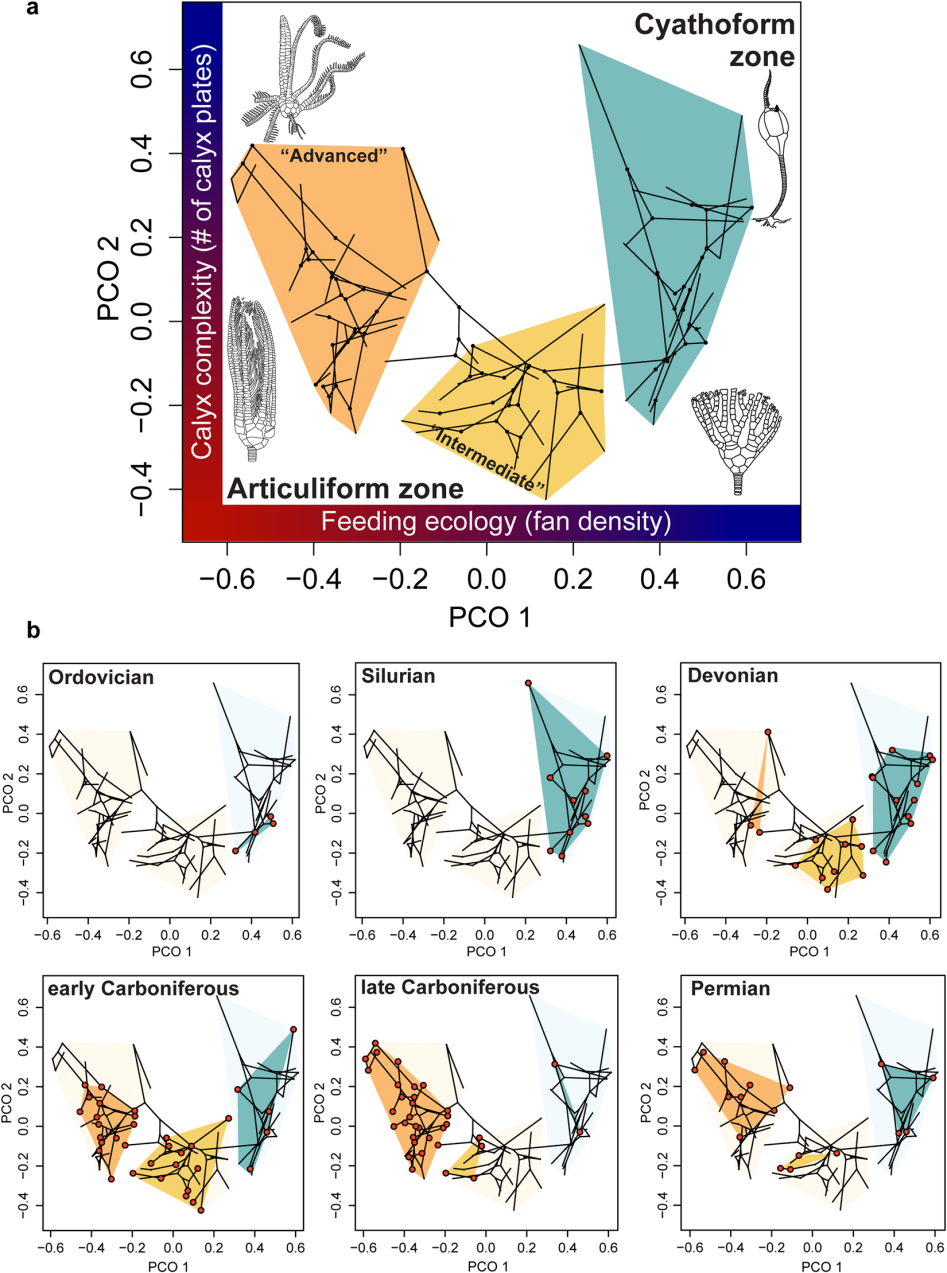
Phylomorphospace and adaptive zone occupation. (**a**) Results of principal coordinate analysis conducted on the entire matrix with taxa joined according to phylogenetic relationships implied by the MCC tree. Red colors on axes represent higher values of calyx complexity and filtration fan density, blue colors indicate lower values. The first two PCO axes summarize 58.63% of the variation. Representative taxa are shown to exemplify regions of crinoid morphospace. All drawings modified from ref.^42^. (**b**) Time series of adaptive zone occupation. Taxa occurring within time bins are shown as red circles. Cyathoform taxa are distributed in the blue region on the right hand side of PCO1, whereas taxa within the two articuliform subzones are in the center and left (articuliform subzone names *sensu* refs^30,56–58^).

The early history of eucladid crinoid evolution predominantly reflects patterns of cyathoform diversification. The cyathoform radiation is characterized by decelerating rates through time (Spearman’s *Rho* = −0.930, *P* < 0.001) (Supplementary Information, Fig. S7), consistent with expectations of an early burst model. In contrast, variation in rates after the Devonian is most strongly associated with articuliforms. A small but non-significant rise in mean rates is coincident with the gradual assembly of the articuliform body plan during the Devonian (Fig. 1), and rates of change within the subclade are higher early in their history than in subsequent intervals (Supplementary Information, Fig. S7). However, long-term rates of change among articuliforms show no evidence for a secular trend (Spearman’s *Rho* = 0.042, *P* = 0.873), and the highest rates of evolution occur much later in their history (Supplementary Information, Fig. S7). Thus, rates of morphologic evolution characterizing articuliform diversification do not support an early burst pattern of phenotypic diversification despite substantial morphologic innovation and expansion of morphospace during the Devonian (Fig. 2).

A morphospace analysis of the complete dataset shows disparity among articuliform crinoids is much greater than among cyathoforms, especially when patterns of morphospace occupation are evaluated over time (Fig. 2). Cyathoform crinoids rapidly expanded in morphospace during their explosive Ordovician-Silurian radiation and reached their maximal disparity during the Silurian. Although cyathoforms continued to diversify throughout the Palaeozoic, they never expanded into new regions of morphospace outside those that originally evolved during the early Palaeozoic. Thus, the early history of cyathoform evolution is consistent with an early burst-type pattern and adaptive radiation; whereas their subsequent diversification during the middle to late Palaeozoic is more consistent with increased constraints that prevented expansion into unexplored regions of morphospace.

In contrast, articuliforms increased in disparity during the Devonian and reached peak morphologic diversity during the early Carboniferous, occupying vast regions of morphospace spanning two adaptive subzones (Fig. 2, Supplementary Information). The distribution of crinoids in morphospace was highly asymmetric during the late Carboniferous, as the “advanced”^30^ articuliform subzone became saturated and very few regions outside this subzone were occupied. However, taxa within this subzone diminished in importance by the Permian. A broad distribution in morphospace coupled with preferential extinction of intermediate forms ultimately led to the Permian being the time of maximal morphologic disparity in the Palaeozoic radiation of eucladids^31,32^.

Alternative models of clade diversification make testable predictions regarding the expected distribution of morphologic rates, disparity, and patterns of lineage diversification (Supplementary Information, Fig. S7). For example, if morphologic changes follow a time-homogenous Brownian motion (BM) process, then the expected relationship between taxonomic diversity and morphologic disparity is linear on a log scale^33^. I tested whether eucladid diversification exhibits evidence for temporal shifts in clade-wide dynamics by constructing a time-series of morphologic disparity and genus-level diversity (Supplementary Information, Figs S8–S13), and then comparing the resulting trajectories in Jablonskian “diversity-disparity space”^34^ with inferred rates of morphologic change (Supplementary Information).

**Figure 3:**
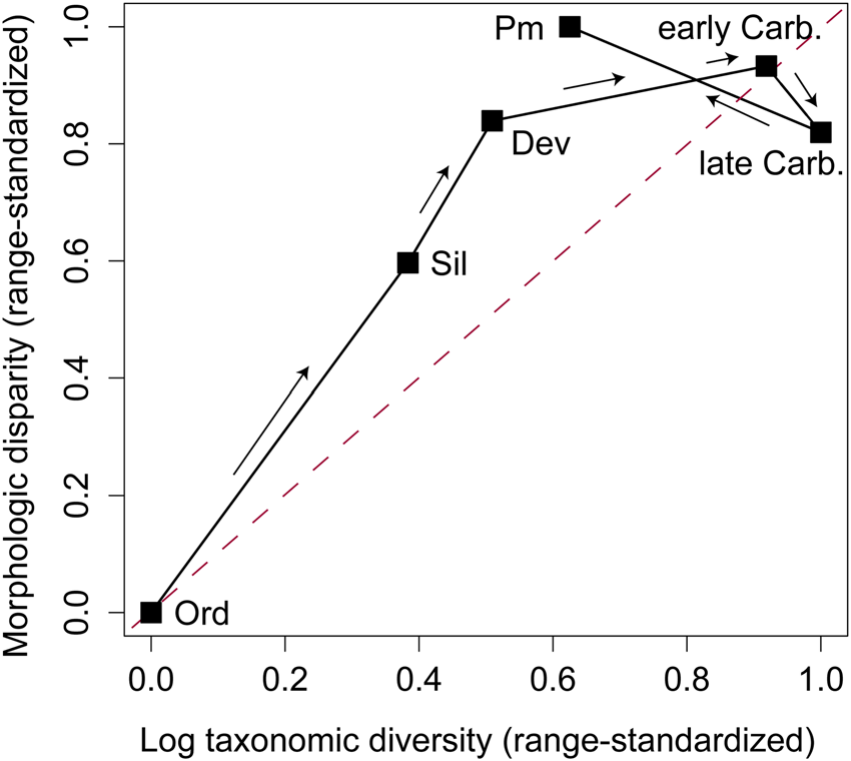
Taxonomic and morphologic diversification in Palaeozoic eucladid crinoids. Taxonomic data are log transformed to produce a linear expectation between taxonomic diversification and phenotypic change under constant rate Brownian motion. Disparity and diversity values are range standardized using [(*x*_*i*_ − min[*x*])/(max[*x*] − min[*x*])]. The red line implies concordance between diversity and disparity^33,34^. If points fall above the dotted red line, disparity is outpacing diversification; whereas, points falling below the line indicate diversification is outpacing disparification. Error bars are not shown, but the Silurian and Devonian are significantly above the 1:1 line and late Carboniferous falls significantly below it (Supplementary Information, Fig. S13). Ord = Ordovician, Sil = Silurian, Dev = Devonian, Carb = Carboniferous, and Perm = Permian.

Diversity and disparity both rapidly increase from the Ordovician through the Devonian and rise significantly above the 1:1 diagonal (Fig. 3, Supplementary Information, Fig. S13). The end-Ordovician mass extinction dramatically altered the structure of crinoid communities^35^, but it is not clear how the extinction may have affected subsequent diversification. Net taxonomic diversification fell dramatically during the Late Ordovician (Supplementary Information, Fig. S3), but the overall taxonomic diversity trajectory shows a nearly constant rate increase from the Ordovician through the end of the Silurian (Fig. 3, Supplementary Information, Fig. S10). It is possible the end-Ordovician mass extinction played a role in sustaining rates of morphologic evolution for such geologically long intervals. For example, the opening of niche space that followed the mass extinction may have allowed the early Palaeozoic radiation to continue in a protracted diversification. Alternatively, the extinction event may have cleared crinoid ecospace and led to a post-extinction adaptive radiation^32^. If so, then sustained elevated rates during the early Palaeozoic reflects two contiguous diversification events rather than a single protracted radiation.

Post-Silurian patterns are suggestive of increasingly complex dynamics, with morphologic rates decoupled from patterns of taxonomic diversification and adaptive zone occupation. Despite the considerable expansion into new regions of morphospace during the Devonian (Fig. 2), the increase in disparity was not accompanied by a significant increase in morphologic rates. Instead, rates of morphologic evolution do not differ statistically from background for much of the Devonian (Fig. 1) (Materials and Methods). Further, the Late Devonian marks the only interval in Palaeozoic crinoid diversification with evidence for significantly low rates of character change (Fig. 1). Thus, morphologic innovation associated with the expansion to the articuliform adaptive zone did not result in an “early burst”-like peak in morphologic rates^9^. Instead, crinoid diversification from the Devonian to early Carboniferous is more similar to a diffusion-like process with expanding boundaries. Brownian motion diffusion through morphospace would be expected to result in increased disparity even if morphologic change was occurring at a low, constant rate^36^. Thus, the Devonian increase in disparity did not result from elevated rates of change, but instead from a shift in the underlying mode of morphologic evolution associated with the expansion into the articuliform adaptive zone.

The Carboniferous features a dramatic increase in taxonomic diversity, marking the so-called “Age of Crinoids”^37^ (Supplementary Information, Fig. S10). Notably, taxonomic diversification was most dramatic among the Articuliformes during this interval. The macroevolutionary lag^38^ between the assembly of the articuliform body plan and their subsequent taxonomic diversification may be explained, in part, by the late Devonian global collapse of reefs. The demise of coral-stromatoporoid dominated reefs facilitated a major environmental transition from abundant, more restricted rimmed carbonate platforms to widespread open ramp settings in epicontinental seas, resulting in a substantial increase in habitat space favorable for crinoids^37^. Global geographic expansion of crinoids during the early Carboniferous may have led to increased speciation propensity^39^, resulting in a marked net increase in taxonomic diversity^37^ (Fig. 3). Although disparity slightly increased during the early Carboniferous, morphospace became more tightly clustered within articuliform subzones (Fig. 2, Supplementary Information Fig. S14). Rates of evolution may have continued to follow a Brownian motion-like process, but with an increasing role for constraint in morphologic diversification rather than unbounded diffusion.

The rise in morphologic rates of evolution during the late Carboniferous is coincident with a rapid increase in taxonomic diversity (Fig. 3, Supplementary Information, Fig. S10), culminating in maximal genus-level diversity and the only post-Silurian rate peak supported by likelihood ratio tests (Fig. 1). However, the late Carboniferous did not exceed the early Carboniferous in net taxonomic diversification (Supplementary Information, Figs S3 and S10), which suggests the diversity peak is a result of increased taxonomic longevity in addition to elevated origination. Episodic, elevated rates can result from early burst-like dynamics when a single lineage subsequently undergoes adaptive radiation^9,13^. However, rates inferred from Bayesian morphologic clock analyses instead reveal multiple lineages were evolving at high rates during the late Carboniferous (Fig. 1), and is associated with an overall decrease in disparity despite statistically elevated rates of character change (Fig. 3). Thus, late Palaeozoic diversification was dominated by multiple subclades evolving within an increasingly narrow region of morphospace contained within a single articuliform subzone (Fig. 2).

Multiple clades rapidly evolving similar morphologic designs during the late Carboniferous is suggestive of a shared response to similar ecologic or selective pressures. For example, Palaeozoic crinoids exhibit a striking temporal trend in calyx plate reduction over time^40,41^. This paedomorphic trend is most pronounced among Carboniferous taxa and is correlated with a reduction in body size^41^. Paedomorphosis in crinoids is linked with shifts in life history as an adaptive response to rapidly fluctuating environmental conditions and habitat disturbance on a global scale associated with geologic change within intracratonic basins^40,41^. Constrained diversification within a limited, circumscribed region of morphospace is consistent with a macroevolutionary Ornstein-Uhlenbeck (OU) model of morphologic diversification. Although phenotypic optima for an OU-like dynamic may have theoretically existed any time after the “advanced” articuliform subzone evolved, the evolutionary attractor during this interval was much stronger than any time before or after the late Carboniferous. This and other instances of replicate radiations within adaptive subzones^22^ strongly suggests that adaptive evolution may result in patterns of constrained phenotypic diversification during instances of ecologic radiation^15^.

If these results are sufficiently general to apply to other clades and times, then the extreme heterogeneity observed in Palaeozoic crinoid evolution strongly suggests that fitting commonly used models of phenotypic evolution to phylogenies of extant species does not adequately capture the shifting dynamics of evolutionary radiations, especially those spanning timescales where extinction and/or recent radiations within subclades have overprinted the signal of a clade’s early history. Similarly, morphologic disparity is a complex “macroevolutionary currency”^34^ influenced by a wide range of possible causal mechanisms, including (but not limited to) rates of morphologic change, extinction selectivity, turnover rates, and the average duration of shared ancestry among taxa^12,13,33,36^. Notably, the processes influencing disparity patterns are often correlated and exhibit non-linear associations^12,34^. Thus, inferences from palaeobiological studies of disparity may be misled in cases where a strict 1:1 relationship between morphospace occupation and rates of phenotypic evolution is assumed. Even if step size transitions derived from disparity curves adequately capture early burst dynamics during early stages of diversification (Fig. 3), the extent to which disparity patterns are subsequently decoupled from morphologic rates remains poorly understood and needs to be furthered tested with other clades. In cases where morphologic changes can be estimated from phylogenetic trees and/or ancestor-descendant pairs, additional comparisons between rates and disparity offer fruitful opportunities to develop a more nuanced understanding of morphologic diversification.

## Materials and Methods

### Morphologic characters and stratigraphic data

I compiled a morphologic data matrix of 92 morphologic characters for 81 species of Palaeozoic eucladid crinoids and one outgroup. The majority of characters included overlap with a previous study^17^, but I excluded any characters with invariant distributions among sampled taxa and included new characters (and character states) to accommodate morphologic variation in middle to upper Palaeozoic crinoids (Supplementary Information, Table S1). To obtain an analytically tractable sample of species for analysis, representative species were chosen from nominal families^16,42^ to capture broad taxonomic, morphologic, and preservational gradients among taxa. In most cases, species sampled were type species of a type genus. However, some geologically older species were sampled, especially when an older species was represented by more completely preserved specimens and was available for coding. Characters were chosen to sample the total range of crinoid body plans without *a priori* consideration of their parsimony-informativeness. All missing or inapplicable characters were coded as “?” (~20% of the cells). The mean number of unscored cells per taxon (18.2%) and per character (16.5%) are both relatively low and similar to one another. Thus, taxonomic and character-based biases introduced by missing data are likely minimal for this dataset. Notably, this dataset is more complete than comparable studies testing phylogeny-based rates of morphologic evolution over geologic timescales^9,23^.

Character-based rarefaction has shown that small subsets of characters comprising the total range of crinoid morphology accurately reflect patterns inferred using larger datasets (~20%)^43^. Thus, the large sample of characters used here likely capture the basic patterns of disparity among crinoids and facilitates estimation of morphologic rates utilizing characters that span the entire body plan. Characters were coded by examining specimens housed in museum collections, including the Smithsonian Institution, Field Museum of Natural History, the Natural History Museum (London), and the Lapworth Museum of Geology. Most species were coded using multiple specimens, such as types and paratypes in their type-series. Additional character data were collected by examining the primary taxonomic literature.

All numerical dates and names for geologic intervals are based on the 2016 global chronostratigraphic timescale^44^. Stratigraphic data constraining species temporal ranges were obtained from an updated version of Webster’s index and bibliography of Palaeozoic crinoids^16^ and the Paleobiology Database (paleobiodb.org). Most analyses consist of data binned to the stage-level and range from the Ordovician (Katian) to Permian (either the Wordian or Wuchiapingian depending on the analysis). However, Silurian data were binned at the series-level in an attempt to make the temporal duration between time bins more comparable and reduce uncertainties arising from stratigraphic correlations of Silurian taxa (see ref.^16^). The Carboniferous Period was subdivided into subperiods: the early Carboniferous (Mississippian) and late Carboniferous (Pennsylvanian). The mean bin duration is 7.2 million years ± 3.8 (S.D.).

### Phylogenetic Analysis

To obtain a distribution of time-calibrated trees, I used a Bayesian fossil tip-dating approach as implemented in MrBayes 3.2.5^45^. A Markov model of character evolution^46^ was used with character coding set to “variable” and a 4-category gamma distribution was incorporated to model rate variation among characters. All characters were treated as unordered. Rate variation among lineages was modeled using the uncorrelated, independent gamma rates (IGR) relaxed morphologic clock model^47^, with an exponential hyperprior (λ = 10) on the variance of the gamma distribution. A truncated Gaussian prior was placed on the base rate of the clock (mean = 0.01, standard deviation = 0.1). The time-varying (piecewise-constant) skyline implementation of the sampled ancestor fossilized birth-death process (SA-FBD) was implemented as a prior distribution on branch lengths^17,48–50^. Thus, SA-FBD parameters were estimated across broad geologic intervals, corresponding to the Late Ordovician-Silurian, Devonian, Mississippian, and the Pennsylvanian-mid Permian. For each interval, SA-FBD parameters were assigned an exponential prior (λ = 10) on net diversification, a Beta(1,1) prior on relative extinction, and a Beta(2,2) distribution on fossil sampling. Because taxon sampling is incomplete at the species-level but broadly covers all major eucladid higher taxa, a diversity-based sampling correction was applied^50^.

Data is lacking for precise numerical estimates of species first-appearances within stage-level bins^16^. Thus, the mid-point of the time bin for each species’ first stage-level appearance was used as a point constraint on occurrence times. Previous work strongly indicates the age for the split between Eucladida and their nearest outgroup occurred during the earliest part of the Katian stage^16,17^. Thus, I placed the tree age prior to correspond to the onset of the Katian. A series of partial and hard topological constraints based on results of Wright^17^ were applied. In addition, preliminary analyses using maximum parsimony^51^ and topology-only Bayesian methods^45^ were used to identify sets of taxa that could be constrained, and clades supported by both methods were given partial clade constraints.

Two Markov chain Monte-Carlo runs with four chains were run for 30 million generations, sampling trees every 3000 steps. MCMC convergence was assessed via MrBayes diagnostics^52,53^ and having obtained effective sample sizes ≥200 for all parameters. Following burn-in, 5,000 time-calibrated phylogenetic trees were retained in the posterior distribution. Although the analysis did not recover any of the order-level taxa listed in the *Treatise on Invertebrate Paleontology* ^42^, the validity of these orders have long been questioned and the results obtained are consistent with previous recommendations for revision of eucladid higher taxa^16,18,30,54–58^ (Supplementary Information, Fig. S15). The recovery of clades descending from well supported nodes have significant taxonomic implications for clarifying relationships and classification among eucladid higher taxa (Supplementary Information).

### Rates of morphologic evolution

Rates of morphologic evolution were inferred using two methods: a maximum-likelihood approach that estimates rates of character changes occurring along branches of time-calibrated trees^59^, and a Bayesian relaxed morphologic clock analysis^45^. To account for uncertainties in evolutionary relationships and divergence times, all rate analyses were performed over random samples of time-calibrated trees from the Bayesian posterior distribution.

Likelihood–based analyses were conducted by first estimating ancestral states of internal nodes from time-calibrated phylogenies using maximum likelihood^59^, and then calculating the number of changes (i.e., number of steps) occurring along a branch divided by the product of time and the proportion of the observed comparable characters^59,60^. Likelihood ratio tests (LRT) were used to test whether rates for a given time bin is significantly higher or lower than those estimated from the remaining pooled bins. The significance level (*α*) for LRTs was set to 0.01. The Benjamini-Hochberg test was used to correct for multiple comparisons and their associated type I errors^60,61^. Because the rates inferred from this method are sensitive to the tree topology and branch lengths, I calculated rates of evolution over a random sample of 100 trees from the Bayesian posterior distribution of time-calibrated trees using wrappers of functions in the R package *Claddis* ^60^. If a time bin contained a threshold of >55% pooled rate estimates with statistically significant (i.e., *P* < 0.01) high or low values across the posterior sample of trees, it was regarded as having positive evidence for rates that deviate from background (Fig. 1).

Rates inferred using a Bayesian relaxed morphologic clock were estimated in MrBayes 3.2.5^45^ using the same tree prior and relaxed morphologic clock models described above for phylogenetic analysis. Time-calibrated branch lengths and rates of evolution were estimated simultaneously. Percent character changes per million years were obtained by taking the median values of rates sampled from the posterior distribution.

### Taxonomic diversification

Taxonomic diversity and diversification rates were estimated using data from two sources: (**1**) from a synoptic database from an updated, synoptic compilation of 505 valid eucladid genera^16^ and (**2**) the Paleobiology Database. For the synoptic database generic diversity was estimated using the first and last appearances recorded in ref.^16^ with the standard range-through assumption. Occurrence data downloaded from the PBDB were obtained by selecting all Late Ordovician (Katian) to end-Permian genera belonging to the Cladida and Articulata (corresponding approximately to the Eucladida in ref.^17^), excluding all taxa that could not be identified to valid genus or species (e.g., “?”, “sp.”, etc.) [downloaded on January 9, 2017]. Additional analyses comparing taxonomic rates, alternative methodological approaches, and dataset comparisons are provided in the Supplementary Information. Uncertainty in the number of genera occurring per bin was estimated using a Monte-Carlo analysis consisting of 1,000 bootstrap replicates.

### Disparity and adaptive zone occupation

Morphologic disparity was calculated as the mean squared distance between species in morphospace. All characters were unordered and equally weighted. Phenetic distances between taxa were calculated using Gower’s coefficient^62^. which rescales distances by the dividing by the number of comparable characters:

1 $${S}_{ij}=\frac{{\sum }_{k=1}^{v}{S}_{ijk}}{{\sum }_{k=1}^{v}{\delta }_{ijk}}$$

where *S*_*ijk*_ equal distances and *δ*_ijk_ is equal to one if a character *k* can be coded for species *i* and *j* and otherwise equal to zero^60^. Temporal variation in morphologic disparity was assessed by assigning taxa to period-level bins and calculating disparity for each interval. Uncertainty in disparity estimates was evaluated using 1,000 bootstrap replicates.

A principal coordinate analysis (PCO) was conducted on the dissimilarity matrix to visualize morphospace and identify ecologically distinct clusters of taxa (Supplementary Information). A phylomorphospace of the MCC tree was projected on species PCO scores distributed along the first two PCO axes, with ancestral state values inferred from ordination scores via maximum likelihood estimation using the R package *phytools* ^63^ (Fig. 2, Supplementary Information, Fig. S9, S18–S19). Other methods exist for creating phylomorphospaces, such as those that first infer ancestral states and subsequently ordinate nodes and tips together^60^. Here, I only use the concept of phylomorphospace to visualize the phylogenetic structure of taxa in morphospace and do not make inferences regarding the morphology of nodes.

PCO1 scores are inversely correlated with filtration fan density (Supplementary Information, Spearman’s *Rho* = −0.772, *P* < 0.001), and therefore provide a proxy for feeding ecology. Lower PCO1 scores indicate denser filtration fans and smaller ambulacral groove widths (Supplementary Information), which relate to food-size selectivity and habitat preference^26,27,64^. PCO2 is inversely proportional to calyx complexity (Supplementary Information, Spearman’s *Rho* = −0.713, *P* < 0.001), where calyx complexity is defined as the number of primary calyx plates in the cup. Calyx complexity is a phenotypic trait linked with life history strategies^40,41^ (Supplementary Information). The spatial distribution of taxa placed within adaptive zones of principal coordinate space (Fig. 2, Supplementary Information, Fig. S14) closely corresponds to previously identified grades of body plan organization in eucladids: cyathoform grade vs. articuliforms, and “primitive”, “intermediate”, and “advanced” calyces^30,40,56–58^.

## Electronic supplementary material

Supplemental Information

Supplementary Info File #1

## References

[1] Simpson, G. G. *Major Features of Evolution* (Columbia University Press, New York, 1953).

[2] Schluter, D. *The Ecology of Adaptive Radiation* (Oxford University Press, Oxford, 2000).

[3] Erwin DH (2007). Disparity: morphological pattern and developmental context. Paleobiology.

[4] Losos JB (2010). Adaptive radiation, ecological opportunity, and evolutionary determinism. Am. Nat..

[5] Harmon LJ (2010). Early bursts of body size and shape evolution are rare in comparative data. Evolution.

[6] Foote M (1994). Morphological disparity in Ordovician–Devonian crinoids and the early saturation of morphological space. Paleobiology.

[7] Wagner, P. J. “Paleontological perspectives on morphologic evolution” In *Evolution since Darwin: the first 150 years* (Sunderland, MA, Sinauer, 2010), pp. 451–478.

[8] Hughes M, Gerber S, Wills MA (2013). Clades reach highest morphological disparity early in their evolution. Proc. Natl. Acad. Sci..

[9] Hopkins MJ, Smith AB (2015). Dynamic evolutionary change in post-Paleozoic echinoids and the importance of scale when interpreting changes in rates of evolution. Proc. Natl. Acad. Sci..

[10] Foote M (1996). Ecological controls on the evolutionary recovery of post-Paleozoic crinoids. Science.

[11] Wagner PJ (1995). Testing patterns of evolutionary constraint hypotheses with early Paleozoic gastropods. Paleobiology.

[12] O’Meara BC, Ané C, Sanderson MJ, Wainwright PC, Hansen T (2006). Testing for different rates of continuous trait evolution using likelihood. Evolution.

[13] Slater GJ (2015). Not so early bursts and the dynamics nature of morphologic diversification. Proc. Natl. Acad. Sci.

[14] Slater GJ, Pennell MW (2014). Robust regression and posterior predictive simulation increase power to detect early bursts of trait evolution. Systematic. Biol..

[15] Erwin DH (1992). A preliminary classification of evolutionary radiations. Historical Biol..

[16] Webster, G. D. “Paleozoic crinoids, coronates, and hemistreptocrinoids, 1758–2012” (Washington State University, 2015, crinoids.azurewebsites.net).

[17] Wright DF (2017). Bayesian estimation of fossil phylogenies and the evolution of early to middle Paleozoic crinoids (Echinodermata). J. Paleontol..

[18] Wright DF, Ausich WI, Cole SR, Peter ME, Rhenburg EC (2017). Phylogenetic taxonomy and classification of the Crinoidea. J. Paleontol..

[19] Foote M, Raup DM (1996). Fossil preservation and the stratigraphic ranges of taxa. Paleobiology.

[20] Meyer DL, Ausich WI, Terry RE (1989). Comparative taphonomy of echinoderms in carbonate facies: Fort Payne Formation (Lower Mississippian) of Kentucky and Tennessee. Palaios.

[21] Ausich WI, Sevastopulo GD (1994). Taphonomy of lower Carboniferous crinoids from the Hook Head Formation, Ireland. Lethaia.

[22] Slater GJ (2015). Iterative adaptive radiations of fossil canids show no evidence for diversity-dependent trait evolution. Proc. Natl. Acad. Sci..

[23] Close RA, Friedman M, Lloyd GT, Benson RB (2015). Evidence for a mid-Jurassic adaptive radiations in mammals. Curr. Biol..

[24] Ausich WI, Bottjer DJ (1982). Tiering in suspension-feeding communities on soft substrata throughout the Phanerozoic. Science.

[25] Kitazawa K, Oji T, Sunamura M (2007). Food composition of crinoids (Crinoidea: Echinodermata) in relation to stalk length and fan density: their paleoecological implications. Marine Biol..

[26] Ausich WI (1980). A model for niche differentiation in Lower Mississippian crinoid communities. J. Paleontol..

[27] Kammer TW (1985). Aerosol filtration theory applied to Misissippian deltaic crinoids. J. Paleontol..

[28] Ausich WI, Baumiller TK (1993). Taphonomic method for determining muscular articulations in fossil crinoids. Palaios.

[29] Baumiller TK, Messing CG (2007). Stalked crinoid locomotion and its ecological and evolutionary implications. Paleontol. Electron..

[30] Kammer TW, Ausich WI (1992). Advanced cladid crinoids from the Middle Misissippian of the eastcentral United States: primitive grade calyces. J. Paleontol..

[31] Foote M (1999). Morphological diversity in the evolutionary radiation of Paleozoic and post-Paleozoic crinoids. Paleobiology.

[32] Deline B, Ausich WI (2011). Testing the plateau, a reexamination of disparity and morphologic constraints in early Paleozoic crinoids. Paleobiology.

[33] Foote, M. “Models of morphological diversification” In *Evolutionary Paleobiology* 62–86 (University of Chicago press, Chicago, 1996).

[34] Jablonski, D. “Macroevolutionary theory” In *The Theory of Evolution*, S.M. Scheiner, D.P. Mindell, Eds. (University of Chicago Press, Chicago, in press), chap. 17.

[35] Ausich WI, Kammer TW, Baumiller TK (1994). Demise of the Middle Paleozoic crinoid fanua: a single extinction event or rapid faunal turnover?. Paleobiology.

[36] Foote M (1997). The evolution of morphologic diversity. Annu. Rev. Ecol. Syst..

[37] Kammer TW, Ausich WI (2006). The “age of crinoids”: a Mississippian biodiversity spike coincident with widespread carbonate ramps. Palaios.

[38] Jablonski D (2008). Biotic interactions and macroevolution: extensions and mismatches across scales and levels. Evolution.

[39] Simões M (2016). The evolving theory of evolutionary radiations. Trends Ecol. Evol..

[40] Wright DF (2015). Fossils, homology, and “phylogenetic paleo-ontogeny”: a reassessment of primary posterior plate homologies among fossil and living crinoids with insight from developmental biology. Paleobiology.

[41] Kammer, T. W. Paedomorphosis as an adaptive response in pinnulate cladid crinoids from the Burlington Limestone (Mississippian, Osagean) of the Mississippi Valley in *Echinoderm Paleobiology* 177–195 (University of Indiana Press, Bloomington, 2008).

[42] Moore, R. C., Lane, N. G. & Strimple, H. L. Order Cladida Moore and Laudon, 1943 in *Treatise on Invertebrate Paleontology, Part T, Echinodermata 2* (Geological Society of America and University of Kansas Press, Kansas, 1978), T587–T759.

[43] Deline B, Ausich WI (2016). Character selection and the quantification of morphological disparity. Paleobiology.

[44] Cohen, K. M., Finney, S. C., Gibbard, P. L. & Fan, J.- X. The ICS International Chronostratigraphic Chart. *Episodes* **36**, 199–204 (2013; updated 2016).

[45] Ronquist F (2012). MrBayes 3.2: efficient Bayesian phylogenetic inference and model choice across a large model space. Sys. Biol..

[46] Wright AM, Hillis DM (2014). Bayesian analysis using a simple likelihood model outperforms parsimony for estimation of phylogeny from discrete morphological data. PLoS One.

[47] Lepage T, Bryant D, Philippe H, Lartillot N (2007). A general comparison of relaxed molecular clock models. Mol. Biol. Evol..

[48] Gavryushkina A, Welch D, Stadler T, Drummond AJ (2014). Bayesian inference of sampled ancestor trees for epidemiology and fossil calibration. PLoS Comp. Biol..

[49] Gavryushkina A, Heath TA, Ksepka DT, Stadler T, Welch AJ (2017). Bayesian total evidence dating reveals the recent crown radiation of penguins. Sys Biol.

[50] Zhang C, Stadler T, Klopfstein S, Heath TA, Ronquist F (2016). Total-evidence dating under the fossilized birth-death process. Sys. Biol..

[51] Swofford, D. PAUP*: Phylognetic analysis using parsimony (*and other methods). version 4, (Sinauer Associates, Massachusetts, 2002).

[52] Lakner C, van der Mark P, Huelsenbeck JP, Larget B, Ronquist F (2008). Efficiency of Markov chain Monte Carlo tree proposals in Bayesian phylogenetics. Sys. Biol..

[53] Gelman A, Rubin DB (1992). Inference from iterative simulation using multiple sequences. Stat. Sci..

[54] McIntosh GC (2001). Devonian cladid crinoids: families Glossocrinidae Goldring, 1923, and Rutkowskicrinidae new family. J. Paleontol..

[55] Simms MJ, Sevastopulo GD (1993). The origin of articulate crinoids. Palaentology.

[56] Kammer TW, Ausich WI (1993). Advanced cladid crinoids from the Middle Mississippian of the east-central United States: intermediate-grade calyces. J. Paleontol..

[57] Kammer TW, Ausich WI (1994). Advanced cladid crinoids from the Middle Mississippian of the east-central United States: advanced-grade calyces. J. Paleontol..

[58] Kammer TW, Ausich WI (1996). Primitive cladid crinoids from upper Osagean-lower Meramecian (Mississippian) rocks of east-central United States. J. Paleontol..

[59] Lloyd GT, Wang SC, Brusatte SL (2012). Identifying heterogeneity in rates of morphological evolution: discrete character change in the evolution of lungfish (Sarcopterygii, Dipnoi). Evolution.

[60] Lloyd GT (2016). Estimating morphological diversity and tempo with discrete character-taxon matrices: implementation, challenges, progress, and future directions. Biol J Linn Soc.

[61] Benjamini Y, Hochberg Y (1995). Controlling the false discovery rate: a practical and powerful approach to multiple testing. J. Roy. Stat. Soc. B.

[62] Gower JC (1971). A general coefficient of similarity and some of its properties. Biometrics.

[63] Revell LJ (2012). Phytools: an R package for phylogenetic comparative biology (and other things). Methods Ecol. Evol..

[64] Foote M (1994). Morphology of Ordovician-Devonian crinoids. Univ. Mich. Mus. Paleontol. Contrib..

[65] Bather, F. A. The Crinoidea, chapter XI, pt. 3, The Echinodermata in *A treatise on zoology* (ed. E. R. Lankester) 94–204 (1900).

