# Supplementary material for: Phenotypic Innovation and Adaptive Constraints in the Evolutionary Radiation of Palaeozoic Crinoids
Source: Sci Rep. 2017 Oct 23;7:13745. doi: 10.1038/s41598-017-13979-9 (PMC5653864; doi:10.1038/s41598-017-13979-9)
Supplement: Supplementary file 1 — Supplemental Information [file 41598_2017_13979_MOESM1_ESM.pdf]

Supplementary Information for:

Title: Phenotypic Innovation and Adaptive Constraints in the Evolutionary Radiation of Palaeozoic Crinoids

Author: **David F. Wright**

Author Affiliation and contact information: Department of Paleobiology, National Museum of Natural History, Smithsonian Institution, Washington, D.C. 20013, USA.

wrightda@si.edu

Corresponding Author: David F. Wright

Disparity vs. rates of morphologic evolution

The view taken here is that morphologic disparity and phylogeny-based rates of morphologic evolution offer complementary ways to examine morphologic evolution, but emphasize different aspects of phenotypic diversification. For the last several decades, palaeobiologists have tested conceptual models of morphologic diversification by measuring temporal variation in morphologic disparity in fossil lineages[1-2]. Disparity profiles are then contrasted with trends in taxonomic diversity to determine which alternative model best fits the observed trends[3-4]. A common assumption is that fluctuations in disparity primarily reflect underlying rates of morphologic change. Although this prodigious research program has generated valuable insights into the mechanisms of morphologic diversification[1-5], there are several causes for concern as to whether or not disparity measures provide a general context for inferring phenotypic rates of evolution. For example, alternative evolutionary processes with strikingly different underlying rates of morphologic evolution can yield equivocal disparity profiles[6-7]. Moreover, even under simple models of trait evolution, such as Brownian motion, sister clades with different rate dynamics may theoretically obtain identical disparities because of the way trait variation is partitioned among subclades[8].Thus, assessing the rate of morphologic evolution requires the explicit use of phylogenetic trees[7-8]. When combined with time-calibrated phylogenies, disparity estimates can be used to test relationships between rates of change and morphospace occupation[9]. Importantly, disparity is inherently a multidimensional concept, whereas rates are unidimensional. Some disparity metrics are better at capturing dynamics of radiations (e.g., changes in the volume of a clade's morphospace) and others are more appropriate for comparing positions of taxa in morphospace (e.g., "niche competition").

Taxonomic patterns of diversification

Webster's index of Palaeozoic crinoids[10] is the most comprehensive, taxonomically vetted dataset of eucladid crinoids in the literature. Unfortunately, the density of genus-level occurrences currently in the PBDB is not sufficient to take advantage of occurrence-based methods at the scale of this analysis[11]. Thus, neither dataset is presently sufficient to take advantage of occurrence-based approaches at the scale of this analysis. Nevertheless, despite Webster's[10] compilation being more taxonomically complete, taxonomic diversity curves generated from stratigraphic ranges derived from this compilation and the PBDB are broadly similar (Figs. S10, S16). Per-capita rates of taxonomic diversification were estimated using equations presented in ref. 12. Net taxonomic diversification per interval is defined as $p - q$, and taxonomic turnover is q/p (Figs. S3, S10, S16-S17).

In addition to taxonomic rates, the time-series of SA-FBD parameters estimated during phylogenetic analysis can also be used to infer diversification dynamics. These parameters are summarized in Fig. S3. Although differences exist between taxonomic and SA-FBD estimates (e.g., the SA-FBD represent coarse timescale averages of species-lineages whereas the finer resolution taxonomic rates are proxies from higher taxa and subject to additional sampling biases [summarized in ref. 11]), the patterns are similar when palaeontologic estimates are averaged over timescales comparable with SA-FBD estimates (Fig. S3). For example, the median rate of taxonomic turnover for the Devonian Period is 0.73, which is close to the SA-FBD estimate of 0.71.

Phylogenetic lineage diversity was calculated as the median diversity across 500 randomly sampled time-calibrated trees from the posterior distribution using the multiDiv function in *palaeotree*[13]. Uncertainty around the median was characterized using two-tailed 95% upper and lower quantiles[13]. Note that the tips of each tree correspond to species observation times (i.e., "first appearances") and therefore do not reflect taxonomic range durations. Because terminal ranges are excluded from these calculations, this becomes a useful metric of taxon sampling effort rather than an estimate of "true" standing diversity. The analyses of genus-level diversity using the two taxonomic databases described above offer more accurate descriptions of taxonomic diversification.

Morphologic disparity and adaptive zones

Although the disparity trajectory for Palaeozoic eucladids is plotted in Figure 3 of the main text, a time series depicting only morphologic disparity is shown in Fig. S8. Although based on different taxon sampling strategies, discrete characters sampled, and recent updates to homologies, the major features and overall trajectory of this curve are strikingly similar to previous disparity profiles generated for Palaeozoic eucladids (e.g., fig. 39 in ref. 14). These similarities to previous studies suggest the character data presented herein are unlikely to contain artifacts related to biased phylogenetic character sampling and are representative of major features in crinoid form.

To visually inspect morphospace distances between taxa and identify ecologically distinct clusters of morphospace (i.e., adaptive zones, see ref. 15), a principal coordinate analysis (PCO) was conducted on the distance matrix as described in the main text. As a results of missing and/or inapplicable data, PCO analysis of the distance matrix extracted negative eigenvalues, all small in magnitude. One solution to handling negative eigenvalues is to choose an arbitrary number of principal coordinates that best summarize the variation among taxa; whereas another possibility is to add a small constant to non-diagonal entries. If variation among axes differed only in scale, then the range and standard deviation of PCO axes should be linearly correlated[14]. Inspection of all principal coordinate axes with positive eigenvalues reveals the first few PCO axes have a higher ratio of standard deviation to range than subsequent axes (Fig. S18), which indicates the first few PCO axes have a different variation structure than the remaining axes. This is also reflected by comparing eigenvalues with their relative rank (Fig. S19). Based on these plots, I chose to present the main results based on seven dimensions. However, all results and inferences were unaffected by either increasing the dimensionality (n = 10, 20 50, etc.) or by choosing to add a small constant to prevent negative eigenvalues. Moreover, because disparity was calculated herein using the mean squared pairwise Gower distance among taxa in morphospace (rather than ordination-based metrics), the results of all disparity analyses are unaffected by differences in the dimensionality of the PCO analysis.

PCO 1 scores are inversely correlated with filtration fan densities (Spearman's $Rho = -0.772$, $P < 0.001$) (42.29% of variation), where filtration fan density reflects the total number of terminal food-gathering appendages (a function of the presence of pinnules, number of distal arms, and arm type [e.g., uniserial/biserial brachials])[16]. Because filtration fan density is inversely correlated with ambulacral groove width[16-17], PCO 1 could also be interpreted as a proxy for ambulacral groove width. In either

interpretation, PCO 1 scores provide an axis for characterizing crinoid feeding ecology. PCO 2 scores are inversely correlated with the total number calyx plates (Spearman's *Rho* = -0.713, $P < 0.001$) (16.34% of variation). Calyx complexity, defined as the number of plates in the cup, is a significant trait reflecting life-history strategies that have been linked with differential rates of ontogenetic growth, taxonomic longevity, habitat preferences, and paedomorphic evolutionary trends[18-20]. It is important to note that neither filtration fan density nor calyx complexity were traits explicitly used in phylogeny reconstruction. Instead, these ecologic and life-history traits are commonly used in the existing literature on crinoid ecology and ontogeny[16-20] because they reflect many aspects of overall morphologic form.

In summary, the primary basis for delineating adaptive zones in the analyses presented herein are feeding mode and life-history strategies. Although the method used to delineate crinoid adaptive zones herein novel to fossil crinoids, the resulting set of taxa placed within adaptive zones (and subzones) are strikingly similar to those previously recognized based on assemblages of Mississippian cladid crinoid faunas. The spatial distribution of taxa placed within adaptive zones of principal coordinate space (Fig. 2, Fig. S14) closely corresponds to previously identified grades of body plan organization in eucladids: cyathoform vs. articuliform (i.e., "poteriocrine"-like, see ref. 21), and "primitive", "intermediate", and "advanced" calyces[22-25]. In this analysis, "primitive" grade calyces largely correspond to morphologies belonging to taxa currently placed within the Cyathoformes[26], whereas both "intermediate" and "advanced" grade calyces broadly correspond to the adaptive subzones for taxa placed within the Clade Articuliformes (see recommendations for taxonomic revision below).

Eucladid systematics and recommendations for taxonomic revision

Although recent work has illuminated phylogenetic relationships among the Crinoidea[26-28], the phylogeny of Palaeozoic eucladid crinoids remain poorly understood and have not received comprehensive systematic treatment since publication of the crinoid *Treatise on Invertebrate Palaeontology*[21]. The superorder Cyathoformes was erected by Wright et al. (26) to include the three orders contained within the crinoid *Treatise*, with each order placed in *incertae sedis*. Results herein identify two major clades of Palaeozoic eucladids: the Cyathofomes and the Articuliformes. This distinction emphasizes that most 'poteriocrine' taxa within Cyathoformes (*sensu* ref. 26) should be placed within a distinct clade. Herein, the Cyathoformes includes taxa previously included subsets of taxa placed in the orders Cyathocrinida and most taxa previously placed within the Dendrocrinida[26-27]. The clade Articuliformes replaces the Poteriocrinida[21], as the genus *Poteriocrinities* was not found to be closely related to other taxa placed within the Poteriocrinida as defined by the crinoid *Treatise*, a result anticipated by Webster in ref. 10. The Articuliformes are sister to the Cyathoformes and comprise the "transitional" dendrocrines[27, 29] and the majority genera previously placed within the Poteriocrinida (except *Poteriocrinites*, and possibly most genera currently placed within the Poteriocrinidae)[21] (Fig. S15). The name Articuliformes is appropriate because this clade includes members of the stem and crown group Articulata. A more detailed discussion on broader issues in crinoid systematics and classification is currently in prep by the author.

SI Figures

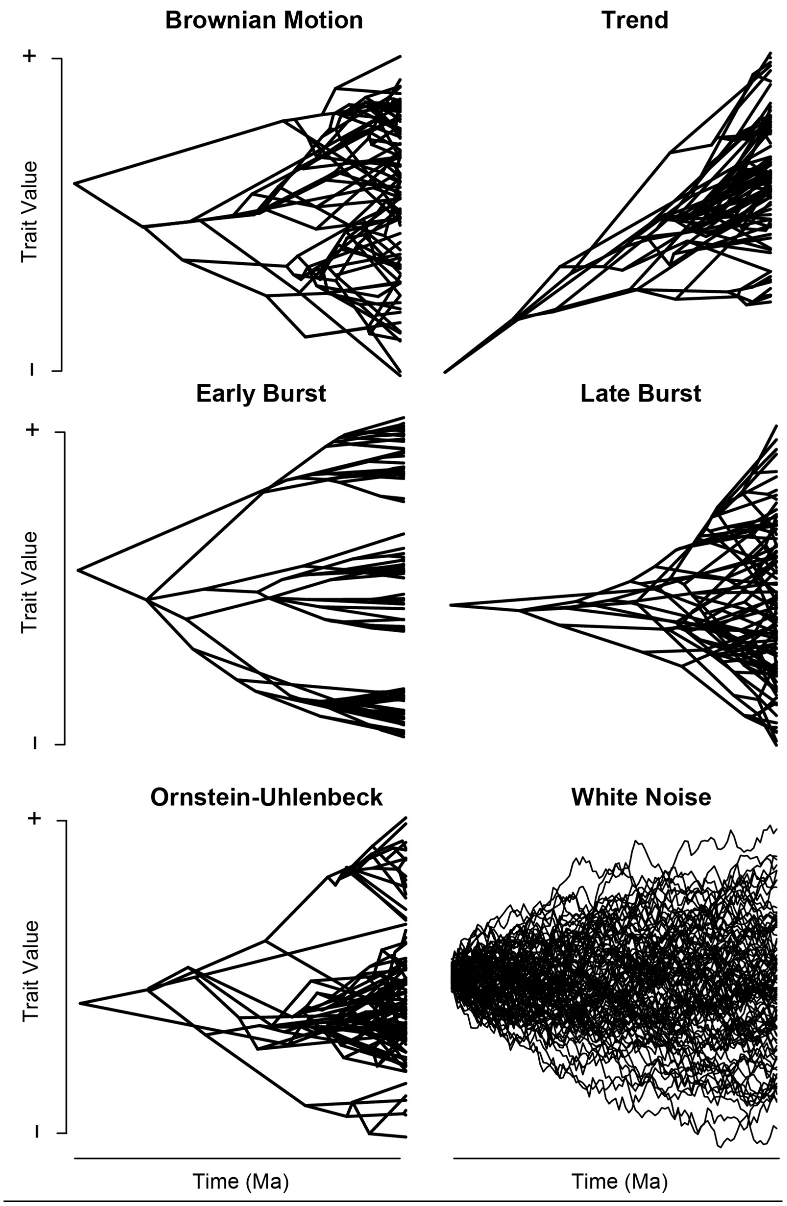

Fig. S1.

Commonly used macroevolutionary models of trait evolution depicted as traitgrams. Models (= "evolutionary mode") differ both conceptually and mathematically, especially with respect to how trait variation accumulates over time.

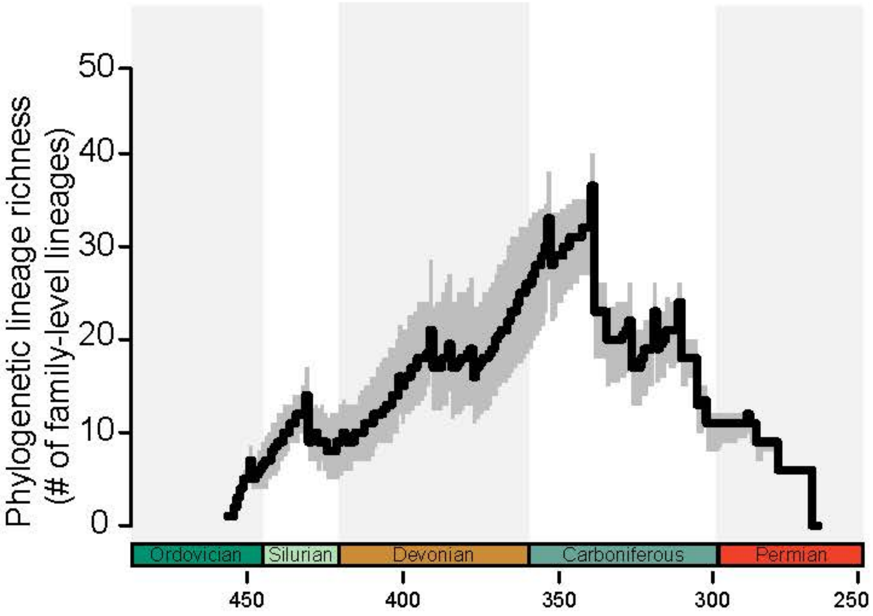

Fig. S2.

Median diversity of sampled taxa for phylogenetic analysis calculated across 500 randomly sampled time-calibrated trees from the posterior distribution. Uncertainty around the median curve is indicated by the shaded region bounded by two-tailed 95% upper and lower quantiles. Note that the estimated peak in rates (Main Text, Fig. 1) do not coincide with times of increased sampling of phylogenetic lineages, thereby making it unlikely that rate estimates can be attributed to taxon sampling strategies.

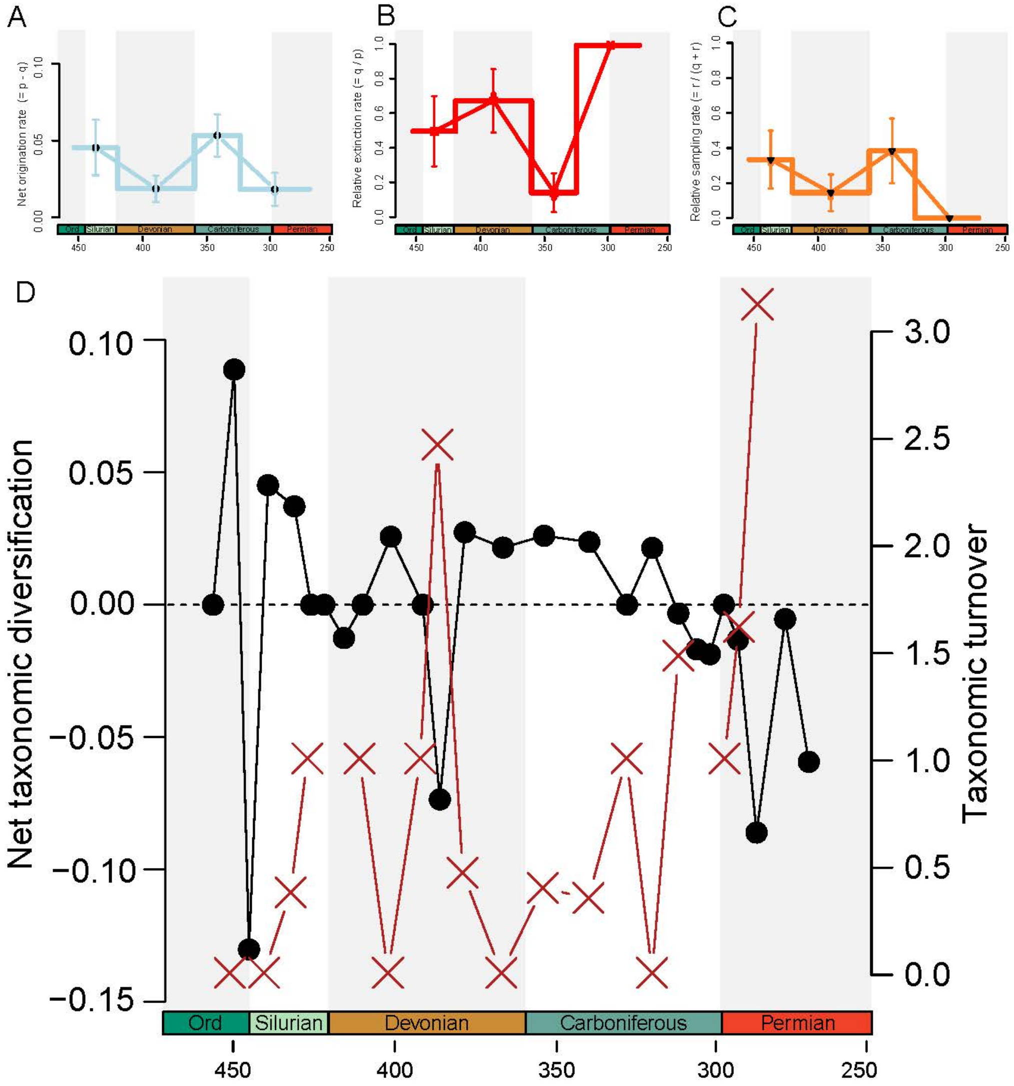

Fig. S3.
Net diversification (A), relative extinction (B), and sampling rates (C) from the Bayesian posterior distribution obtained from phylogenetic analysis. Points indicate median values for all parameter estimates in the posterior distribution. Error bars reflect 95% confidence intervals. The circles (D) represent net taxonomic diversification calculated at the genus-level, whereas the X's indicate per-interval rates of turnover among genera (Materials and Methods).

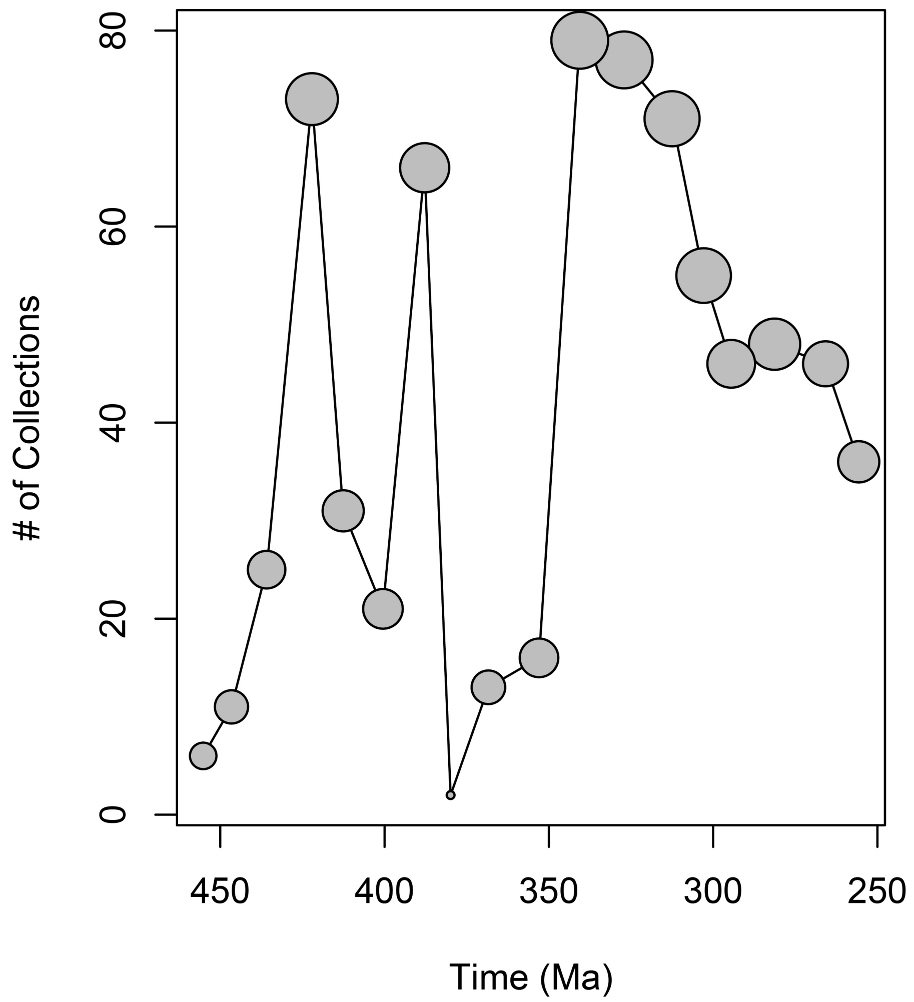

Fig. S4.

Number of collections per-interval contained in the Palaeobiology Database with at least one eucladid crinoid. The size of each point is proportional to the log number of occurrences within its respective 10-million-year time bin. Although sampling intensity fluctuates through time, it is not correlated with inferred evolutionary rates (Results and Discussion).

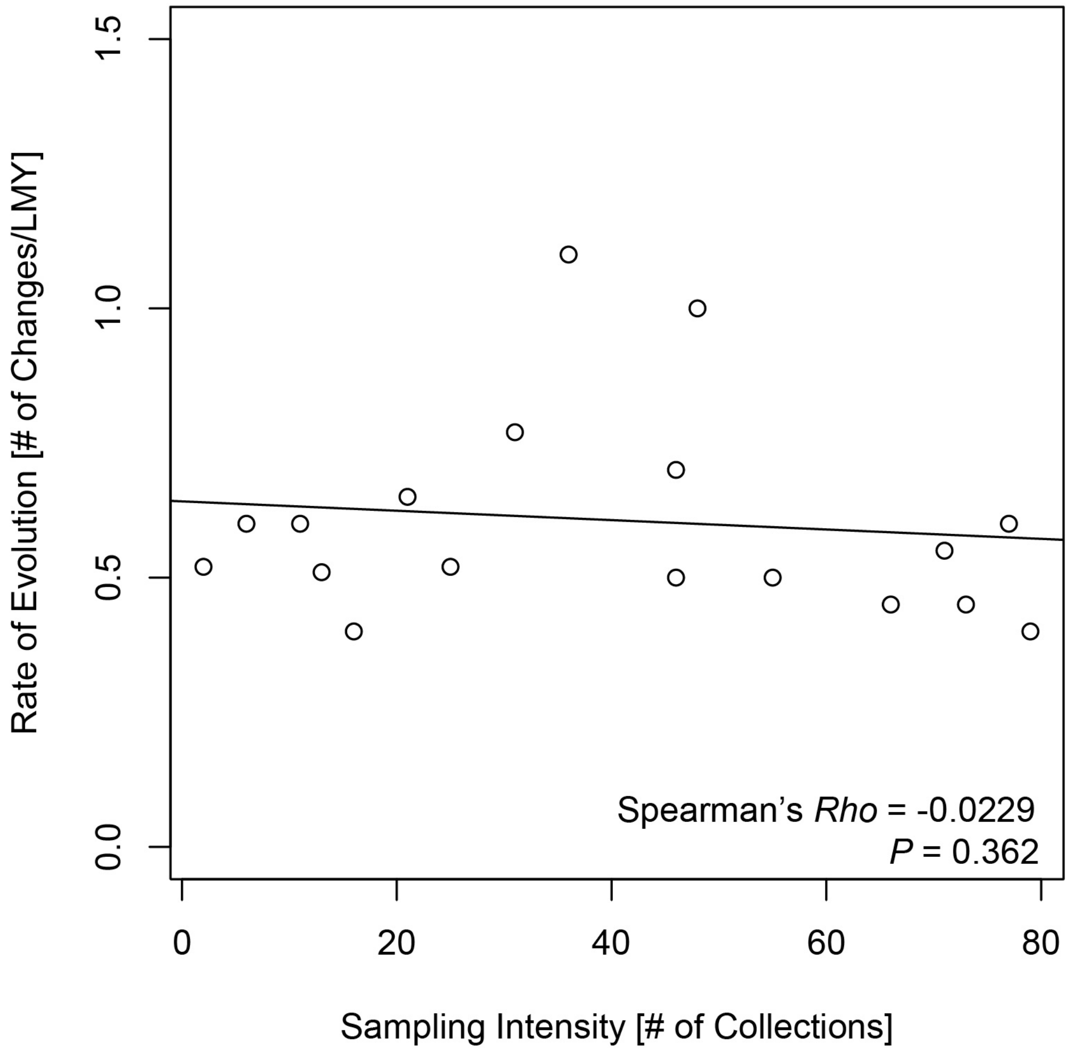

Fig. S5.

Rates of morphologic change are not correlated with sampling intensity, measured as the number of collections per-interval in the Palaeobiology Database containing at least one eucladid crinoid.

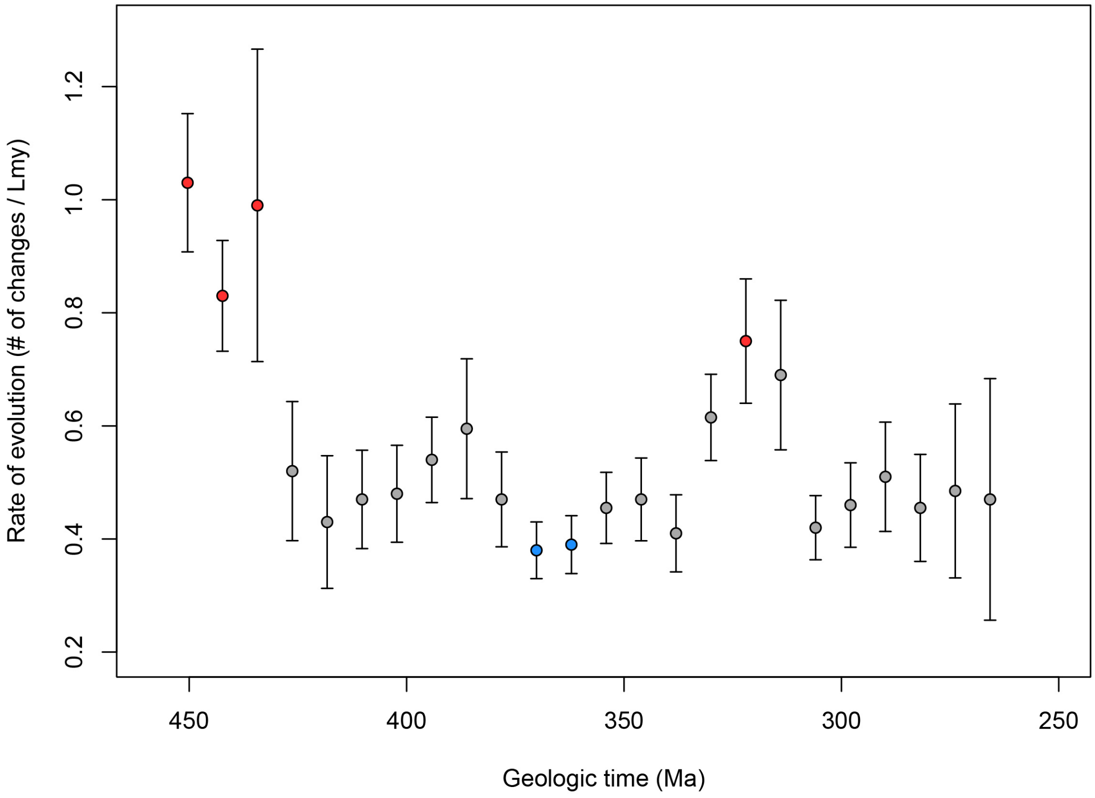

Fig. S6.

Results from maximum likelihood analysis of morphologic rates using equally spaced time bins instead of geologic intervals (i.e., stages and series). Compare with Fig. 1 from the main text.

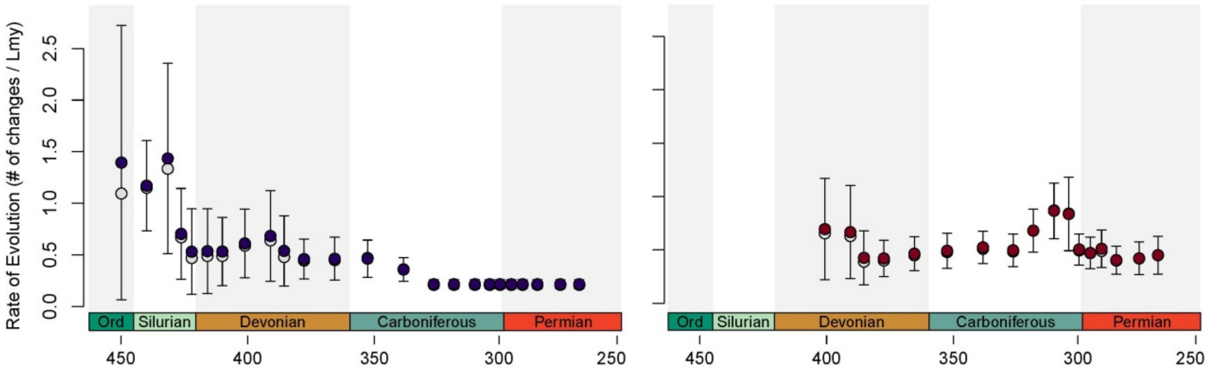

Fig. S7.
Rates of morphologic evolution among the subclades Cyathoformes (left) and Articuliformes (right). Rates were inferred using the maximum-likelihood method over a random sample of 100 time-calibrated trees from the posterior distribution. Colored circles are mean rates and open circles represent medians. Error bars represent 95% confidence intervals.

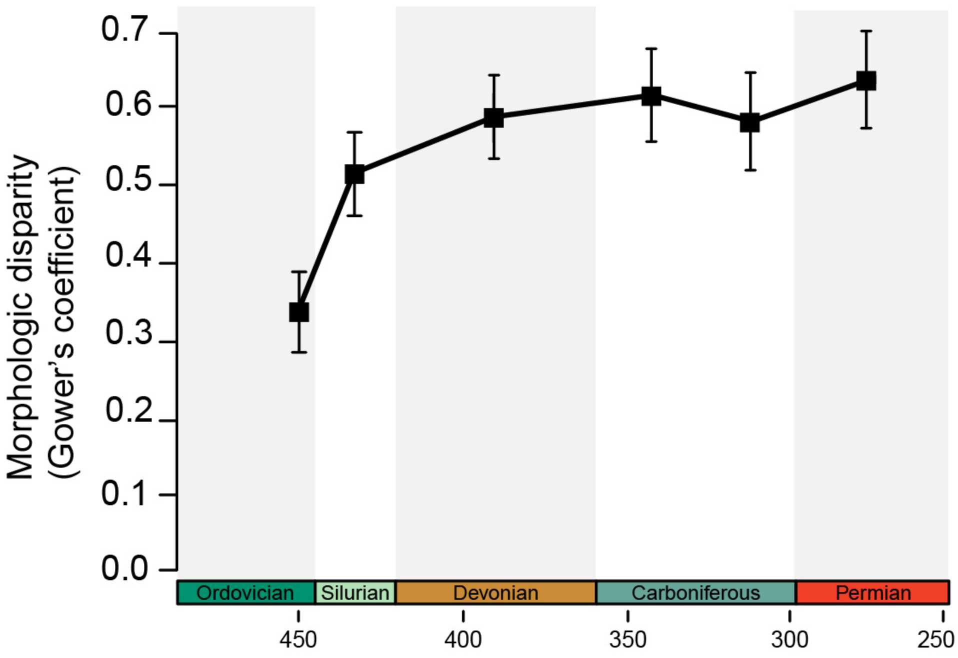

Fig. S8.

Time series of morphologic disparity. Squares indicate the average pairwise distance among species placed in period-level bins. Note the Ordovician point is offset because eucladids originated during the Late Ordovician. Errors bars are 1 standard error based on bootstrap resampling.

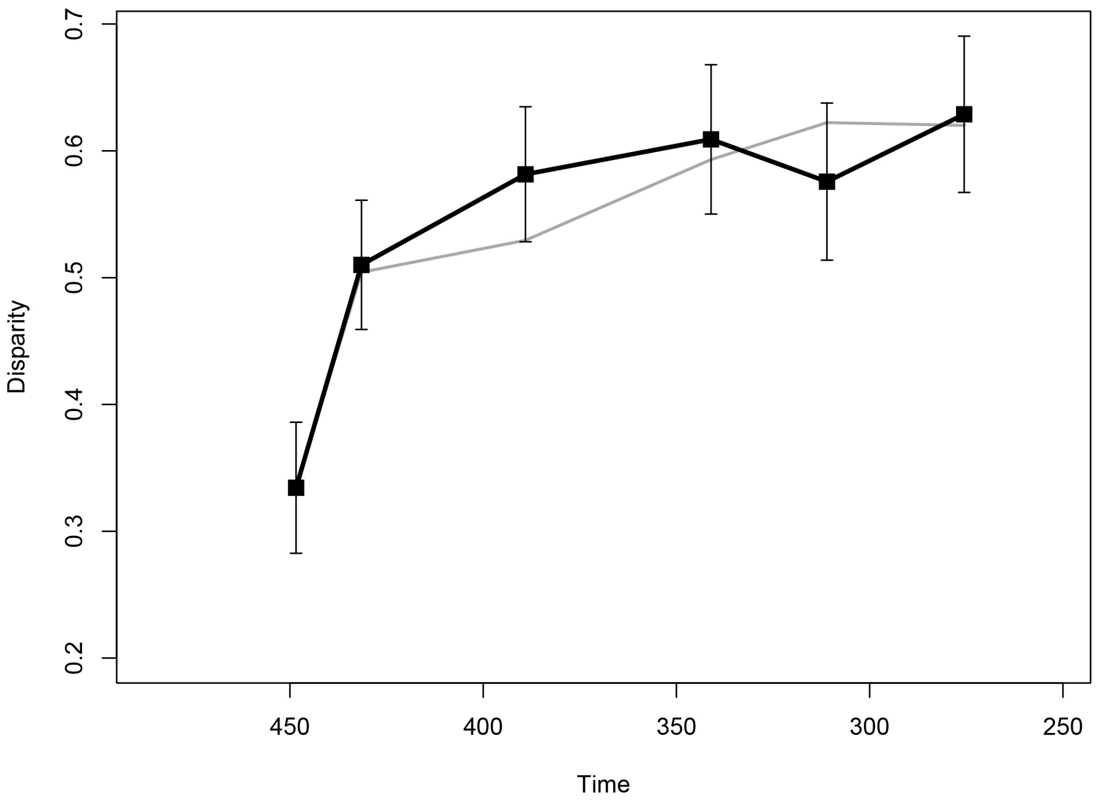

Fig. S9.

Time series of morphologic disparity as in Fig. S8, but with the mean per-period disparity estimated from rarefaction analysis plotted as a grey line.

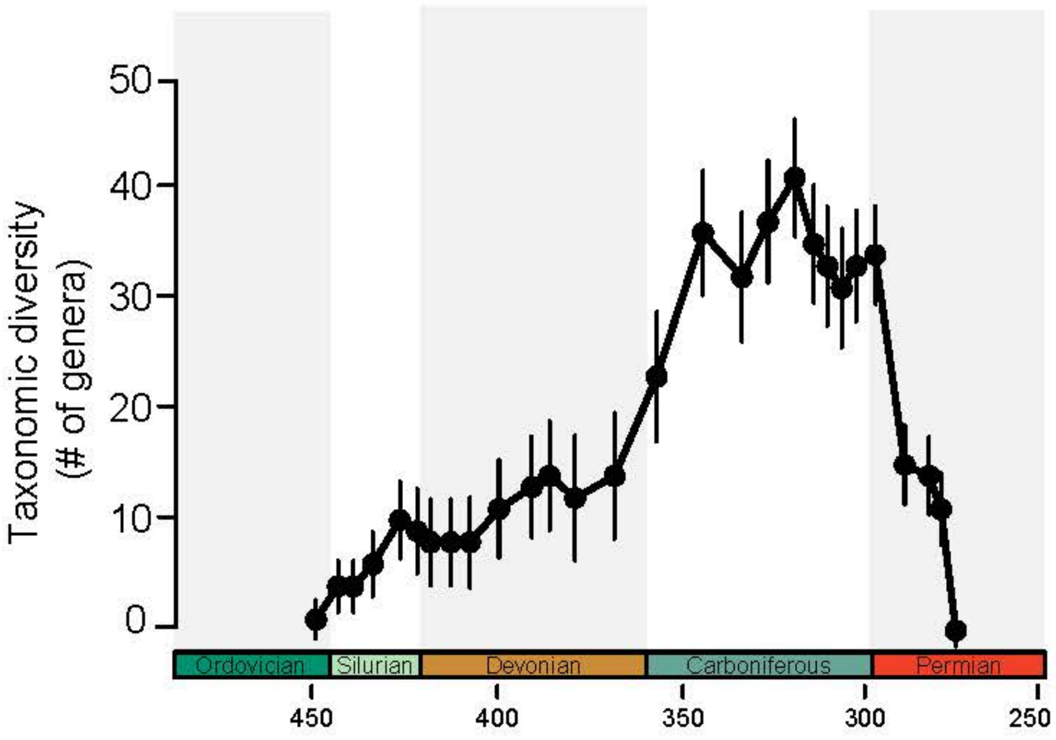

Fig. S10.

Taxonomic diversity of the Eucladida. Circles represent taxonomic diversity, calculated as the number of genera per time bin using taxonomic first and last appearances recorded in Webster's index[16]. Errors bars are 1 standard error based on 1,000 bootstrap replicates.

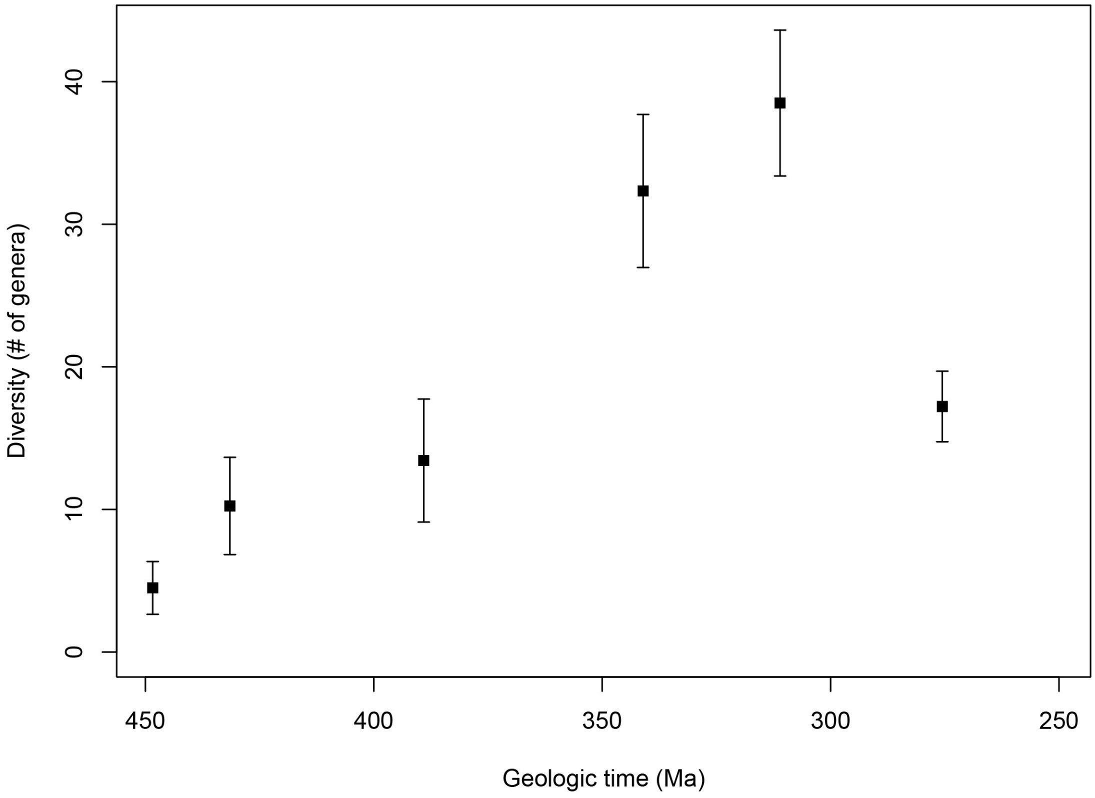

Fig. S11.
Taxonomic diversity of the Eucladida as in Fig. S10, but plotted per-period/subperiod. Errors bars are 1 standard error based on 1,000 bootstrap replicates.

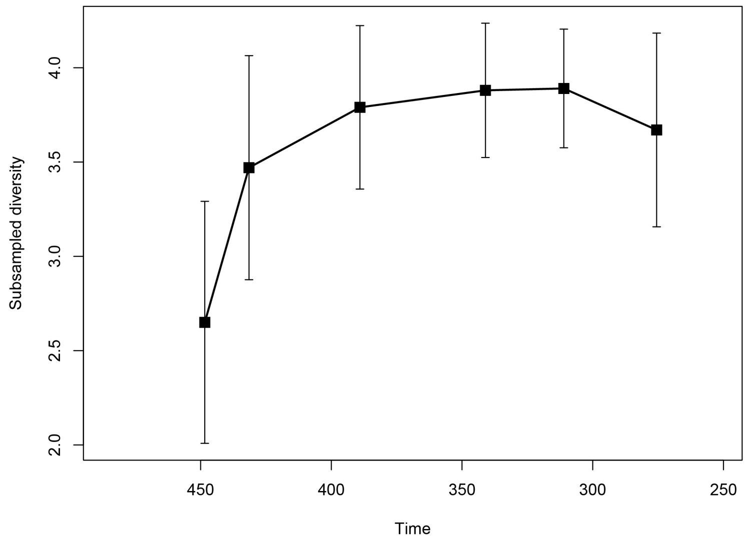

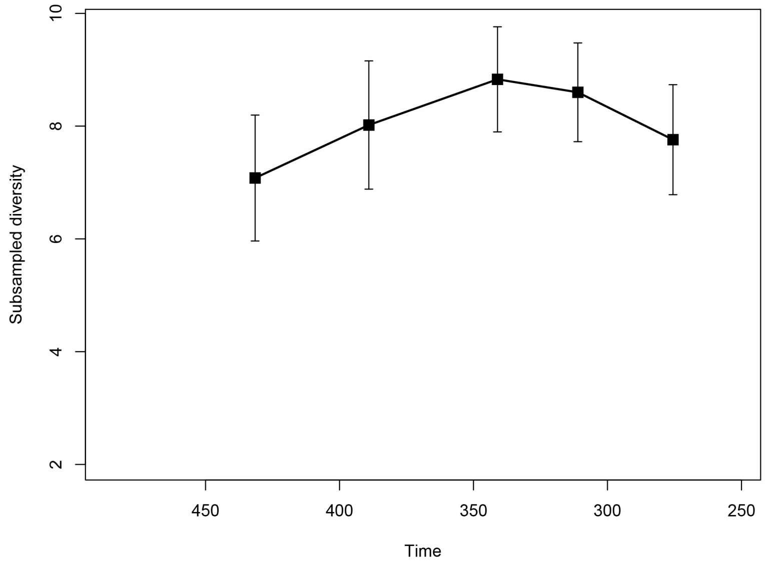

Fig. S12.

Subsampled taxonomic diversity of the Eucladida resulting from rarefaction analysis including (above) and excluding (below) the Ordovician bin; compare with Fig. S11. Errors bars are 1 standard error based on 1,000 bootstrap replicates.

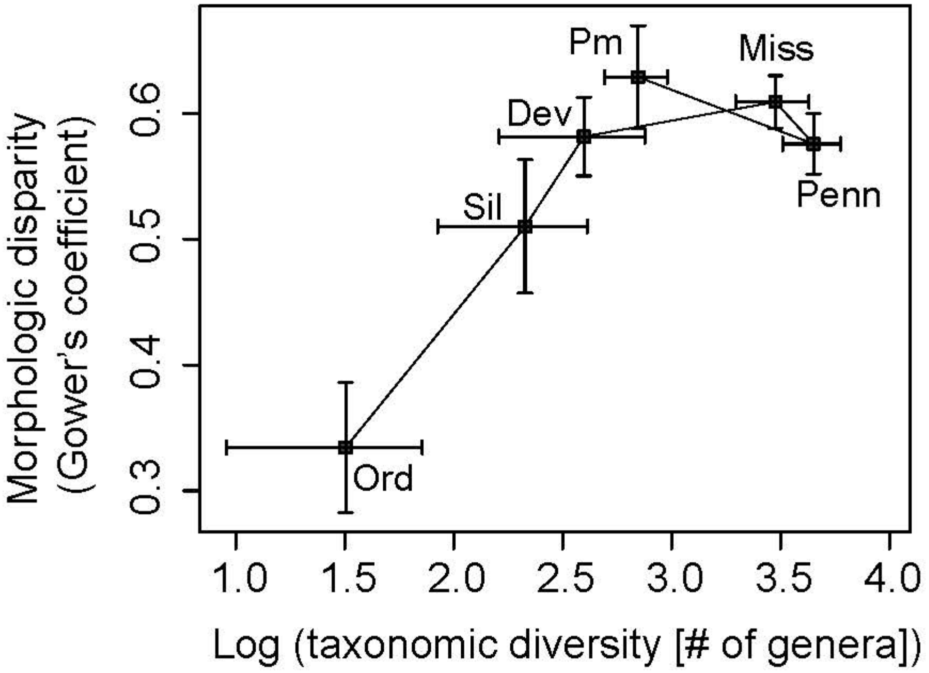

Fig. S13.

Diversity-disparity plot. Similar to Fig. 3 in the main text, but with uncertainty in taxonomic diversity and morphologic disparity plotted in diversity-disparity space. Disparity is measured as the mean squared pairwise Gower distance among species. Taxonomic diversity is shown as the log of generic richness. Both diversity and disparity were range standardized. Error bars are 1 standard error estimated from 1,000 bootstrap replicates.

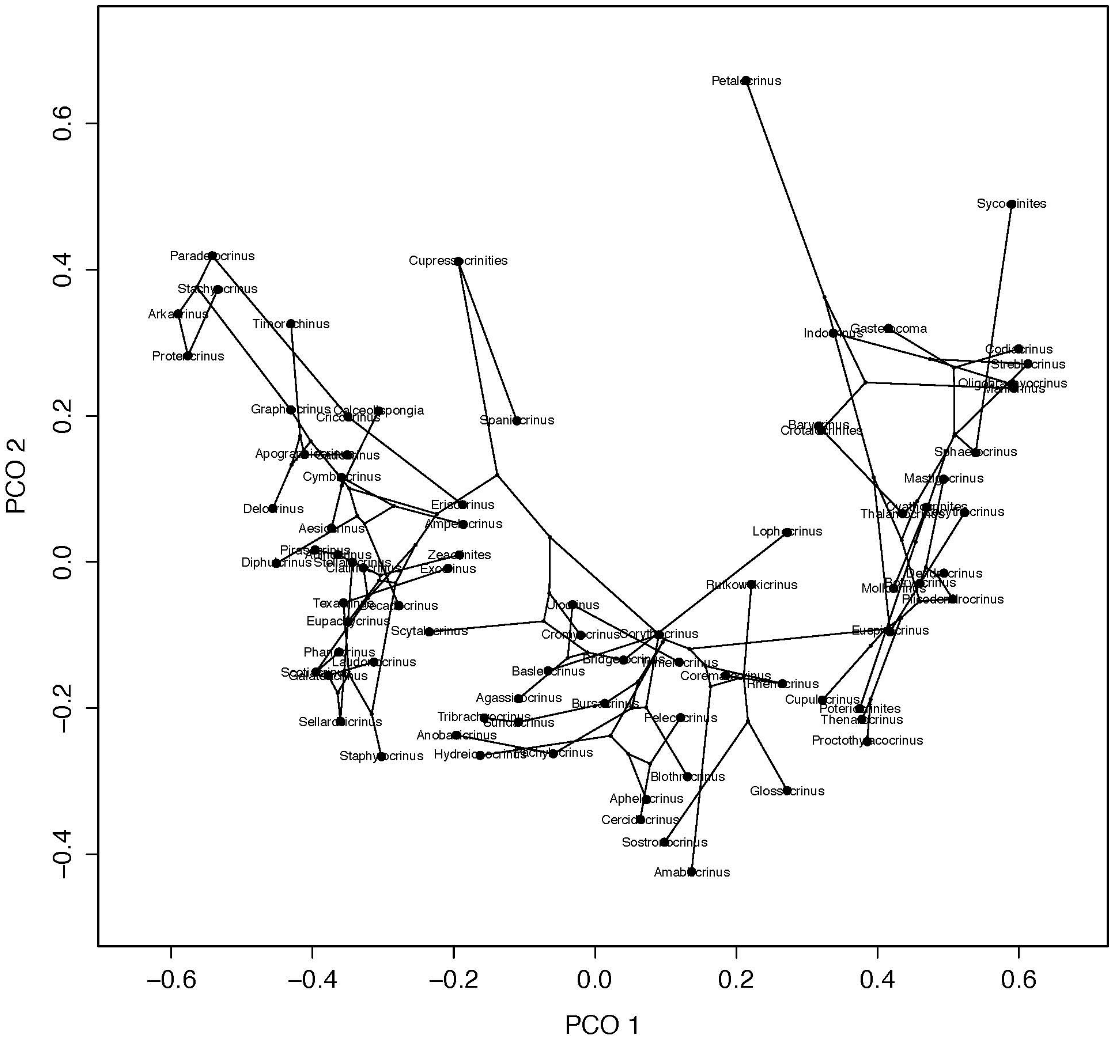

Fig. S14.

Phylomorphospace of the MCC tree from Figure 2 of the main text with tip labels added. The first two PCO axes summarize 58.63% of the variation (PCO 1 = 42.29%, PCO2 = 16.34%).

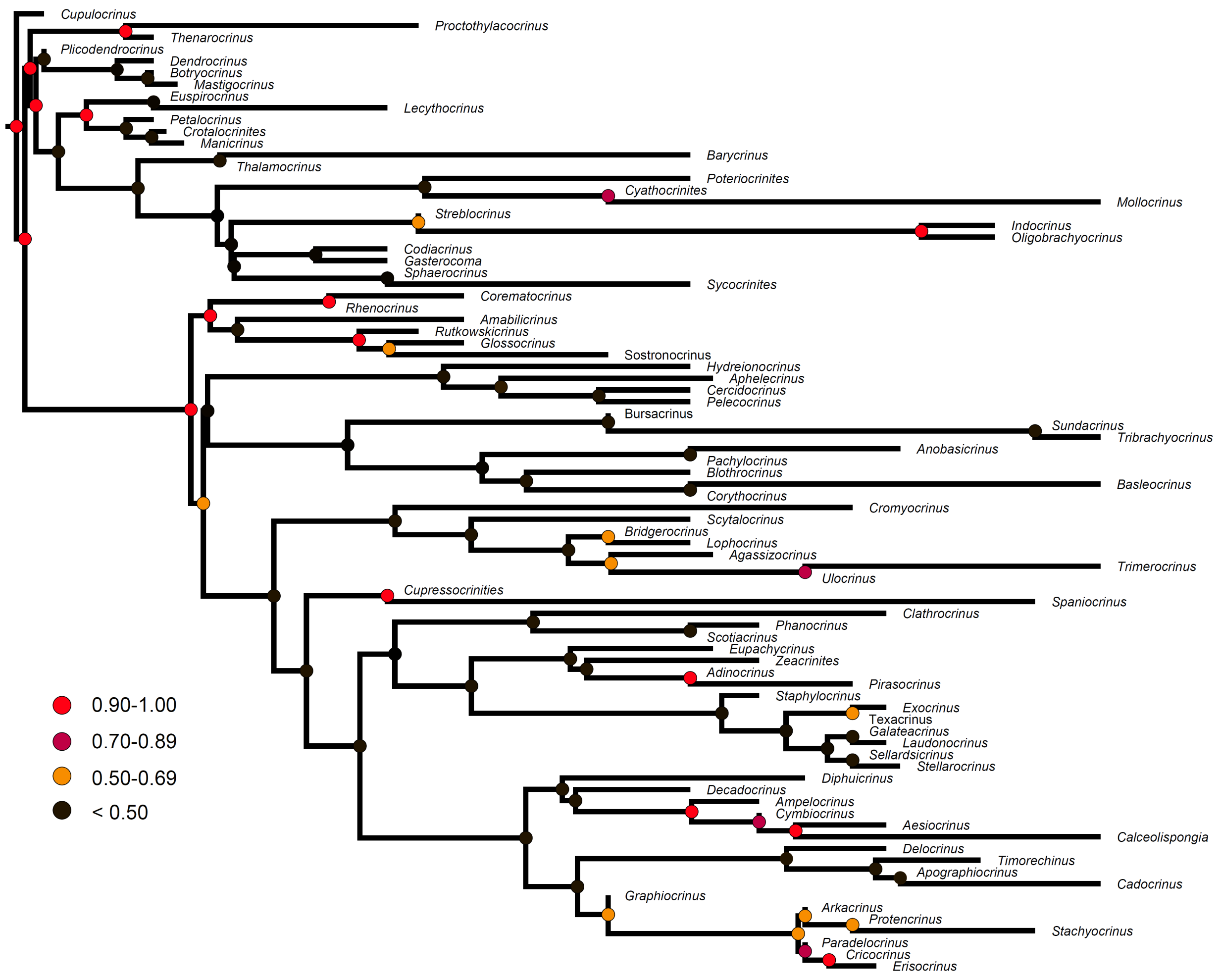

Fig. S15.
Maximum Clade Credibility tree from Bayesian tip-dating analysis. Node support is indicated by posterior probabilities. Basal nodes are well supported, but many more nested nodes are not. However, cyathoform and articuliform clades (main text Fig. 1) are supported with PP = 1. Because the MCC tree contains many poorly supported groups, rate analyses were conducted across a sample of trees from the posterior distribution.

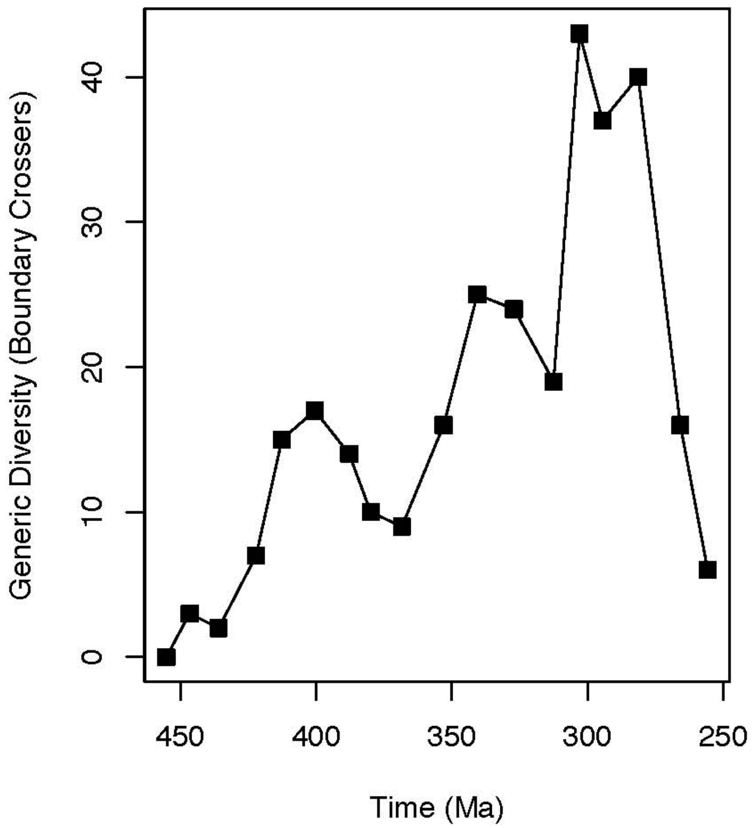

Fig. S16.

Genus-level diversity of eucladids based on occurrences in the Palaeobiology Database, calculated using the boundary crosser method.

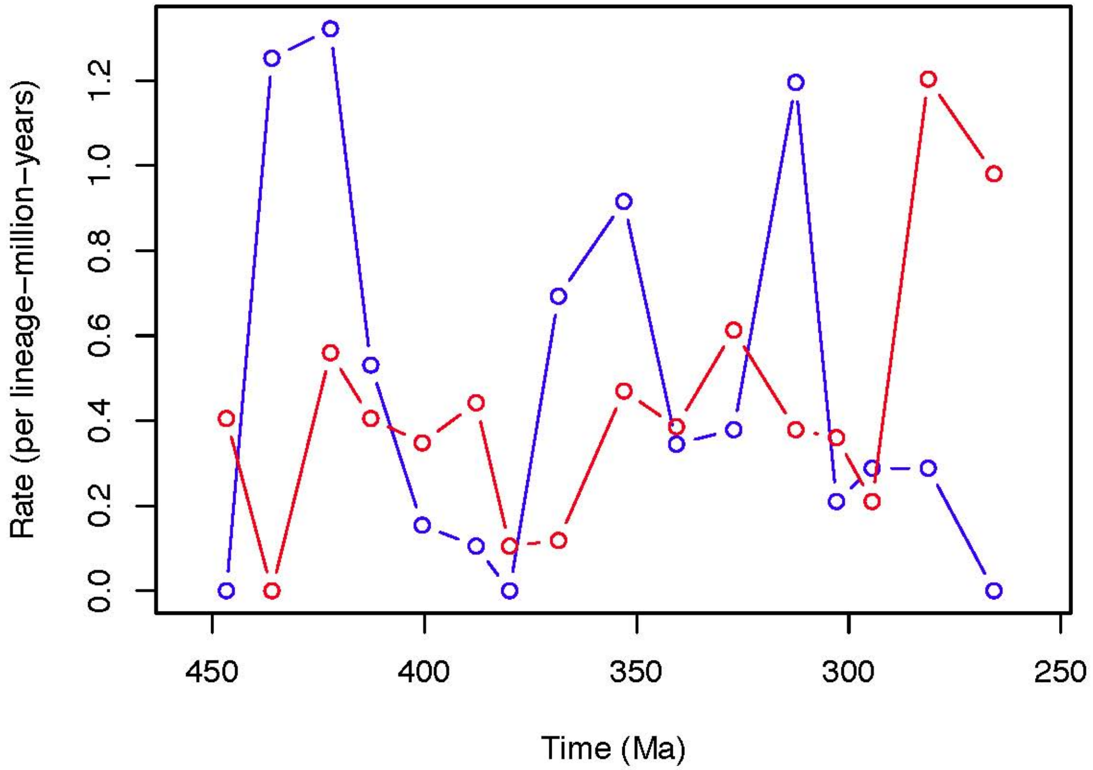

Fig. S17.

Genus-level origination and extinction dynamics of Palaeozoic eucladids based on occurrences in the Palaeobiology Database, calculated using the equations in ref. 12. Origination = blue; extinction = red.

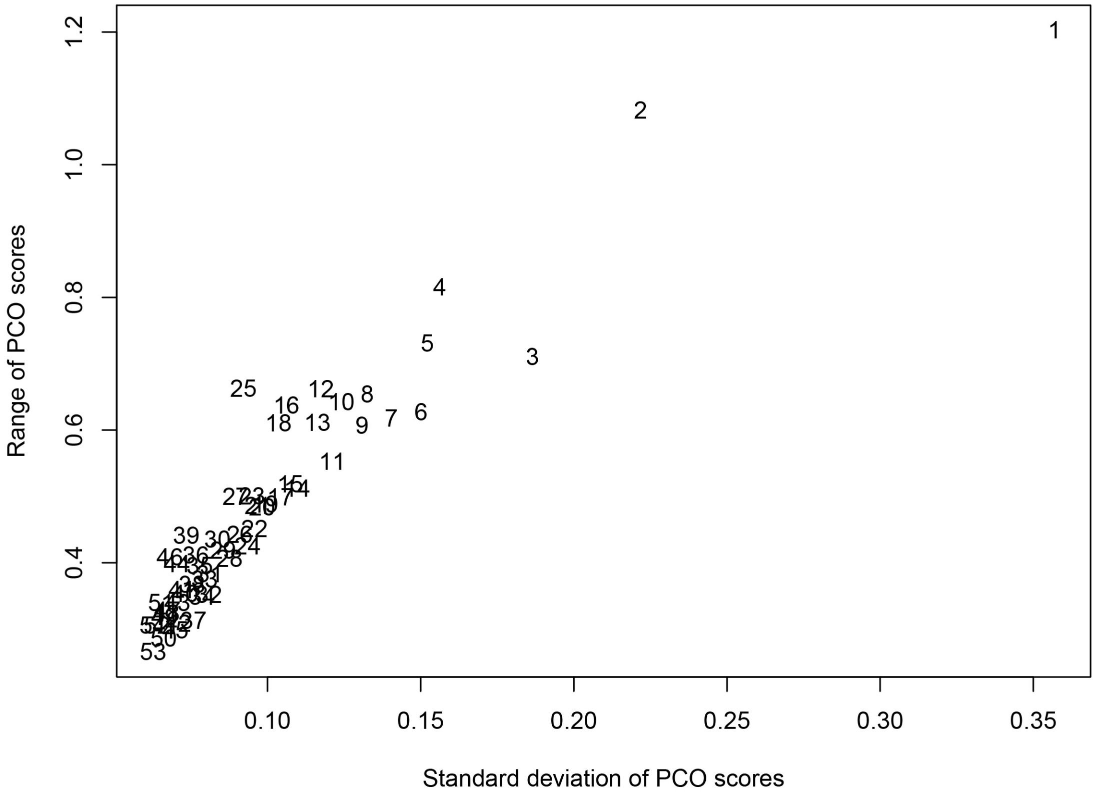

Fig. S18.

Range of PCO scores for each axis vs. their standard deviation. Numbers correspond to their respective PCO axes.

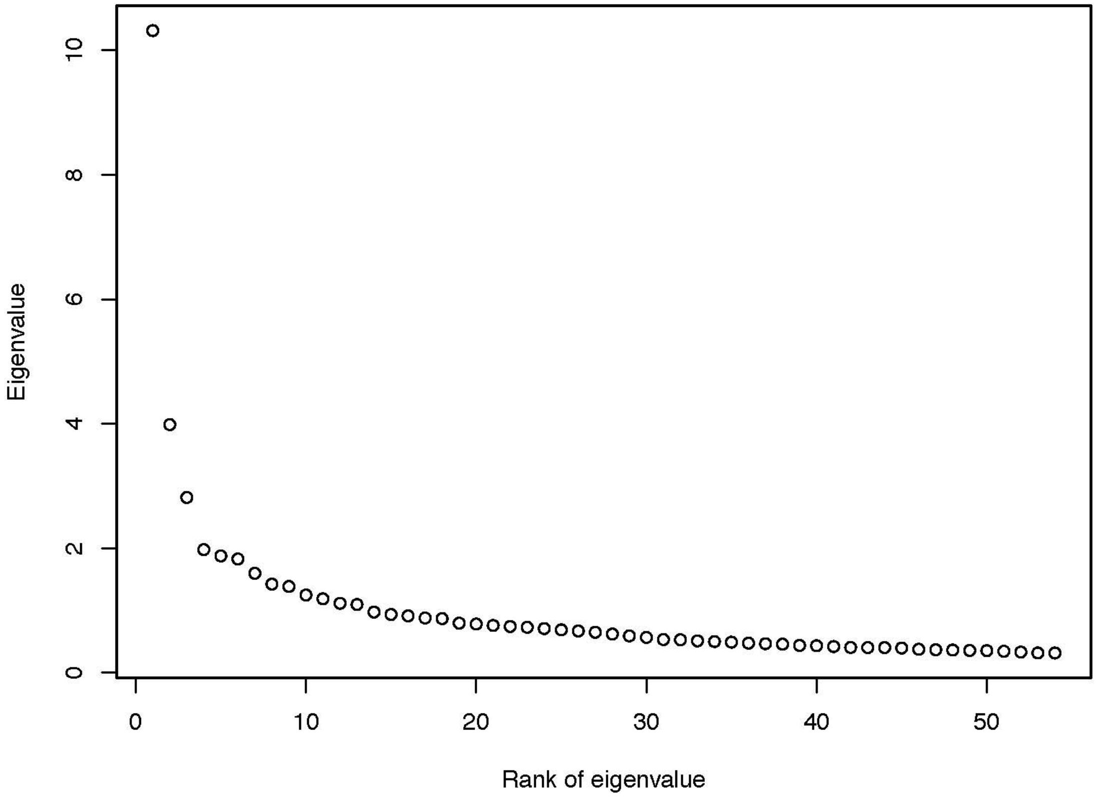

Fig. S19.

Eigenvalue vs. rank of eigenvalues. Similar to Foote's results, the variance structure of the first few PCO axes have

Table S1
Characters used in phylogenetic analysis.

1. Calyx shape ratio (height /width): very high, >1.5 (0); high, 1.5-1.0 (1); intermediate 1.0-0.5 (3); low, 0.5-0.25 (3); very low, < 0.25 (4).

2. Calyx profile: straight sided, cone shape (0); rounded base with widest point at the summit of the calyx (1); rounded base with widest point below the summit of the cup (2).

3. Calyx shape in transverse section: round (0); subpentagonal (1).

4. Calyx radial plate thickness: thin, <25% height or width of plate (0); thick, >25% height or width of plate (1).

5. Calyx plate sculpturing: absent (0); present (1).

6. Type of calyx suturing: non-stellate ridges (0); stellate ridges (1); spinose (2); nodose (3); granulose (4).

7. Outline shape of calyx base: upright (0); broad and flat (1); steeply concave (2).

8. Basal invagination of calyx: absent (0); present (1).

9. Attitude of the infrabasal circlet: all plates visible in side view (0); along base of calyx, neither entirely in a basal concavity nor visible in side view (1); not visible in side view (2).

10. Number of infrabasal plates: five (0); three (1); one (2).

11. Infrabasal plate dimensions: width > height (0); width ~ height (1); width < height (2).

12. Cross sectional shape of the infrabasal plates: straight upwards/flared (0); curved/downflared (1).

13. Attitude of the basal circlet: all plates visible in side view (0); along base of calyx, neither entirely in a basal concavity nor visible in side view (1); not visible in side view (2).
14. Basal plate dimensions: width > height (0); width ~ height (1); width < height (2).
15. Relative sizes of plates in the basal circlet: equal/subequal (0); unequal (1).
16. Cross sectional shape of the basal plates: straight (0); curved (1); bulbous/inflated (2).
17. Interruption in the radial circlet CD interray: absent (0); present (1).
18. Radial plate dimensions: width > height (0); width ~ height (1); width < height (2).
19. Attitude of the radial circlet: (0); along base of calyx, neither entirely in a basal concavity nor visible in side view (1).
20. Cross sectional shape of the radial plates: straight (0); curved (1); bulbous/inflated (2).
21. Largest plate in the calyx: below the radials circlet (0); in the radial circlet (1).
22. Compound/ bi-radial: absent (0); present (1).
23. C-ray radial plate: similar size to other radial plates (0); smaller than other radial plates (1).
24. Radial facet width (*sensu* Webster and Maples 2008): angustary (0); peneplenary (1); plenary (2).
25. Contact type when radial facets extend across the radial plate (Webster 2007): plenary (0); explenary (1); inplenary (2).
26. Radial facet dimensions: width > height (0); width ~ height (1); width < height (2).
27. Adaxial notch on radial facets: absent (0); present (1).
28. Orientation of the radial facets: planate (0); declivate (1); sursumate (2).

29. Radial facet type (*sensu* Webster and Maples, 2008): unifascial (0); bifascial (1); multifascial ‘muscular’ (2); trifascial (3).

30. Transverse ridge on radial facet: absent (0); present (1).

31. Thick entoneural canal on facet: absent (0); present (1).

32. Ligament pit on facet: absent (0); present (1).

33. Crenulated surface proximal to aboral ligamentary fossae: absent (0); present (1).

34. Number of posterior plates partially or completely within the cup: three (0); ≤ three (1).

35. Radianal plate: absent (0); present (1).

36. Visibility of the radianal plate: visible on the exterior (0); cryptic (1).

37. Shape of the radianal plate (upper plate when subdivided): pentagonal (0); tetragonal (1); hexagonal (2).

38. Proximal position of the radianal plate: full width beneath the C-ray radial (0); to the left and below the C radial plate (1); within the radial circlet above the CD basal plate (2).

39. Circlet in most proximal position with the radianal plate: basals (0); infrabasals (1); radials (2).

40. Anal X: absent (0); present (1).

41. Shape of the anal X plate: pentagonal (0); tetragonal (1); hexagonal (2);

42. Position of the anal X plate within the calyx: entirely in the cup (0); partially in the cup (1); entirely above the cup (2).

43. Position of the anal X plate relative to the plates below: above and to the left of the radianal and in contact with the CD basal (0); above and to the left of the radianal

and in contact with the D radial but not the CD basal (1); directly above the radianal and not in proximal contact with the CD basal or the D radial (2).

44. Plates right-lateral to the anal X: in contact with the C-ray radial (0); in contact with other anal plates (1).

45. Right tube plate: absent (0); present (1).

46. Shape of the right tube plate: pentagonal (0); tetragonal (1); hexagonal (2).

47. Position of the right tube plate within the calyx: entirely in the cup (0); partially in the cup (1); entirely above the cup (2).

48. Position of the right tube plate relative to the plates below: above and to the right of the anal X and in contact with the radianal (0); directly above the anal X and not in contact with the radianal (1); above and to the right of the anal X and not in contact with the radianal (2).

49. Plates left-lateral to the right tube plate: anal X and additional anal plates (0); anal X only (1); D-ray radial plate (2).

50. Anal sac: absent (0); present (1).

51. Height of the anal opening above the cup: ≤ half the height of the arms (0); > half the height of the arms and ≤ maximum arm height (1); > than maximum arm height (2).

52. Anus position relative to the crown: near the summit (0); below the summit (1).

53. Anal sac plating: vertical columns, regular plating (0); irregular plating (1).

54. Medial column supporting the anal sac: absent (0); present (1).

55. Anal sac plate sculpturing: absent (0); present (1).

56. Anal sac sculpturing pattern: radiating ridges (0); vertical grooves and ridges (1).

57. Anal sac plate cross section: flat (0); convex (1); spinose (2); plicated (3).

58. Shape of the anal sac: cylindrical (0); tapers distally (1).

59. Pore structures on the anal sac: absent (0); present (1).

60. Anal sac spines: absent (0); present (1).

60. Number of arm openings originating from the calyx: five (0); three (1); one (2).

61. Arrarngement of spines along the anal sac summit: single spine (0); multiple spines (1); roof-forming (e.g., “umbrella” of spines).

62. Arm openings into the calyx: five (0); three (1); one (2).

63. Proximal free arm projection: outward and upward (0); directed vertically (1).

64. Fixed lower brachial plates: absent (0); present (1).

65. Primaxil branching on B-E rays: absent (0); present (1).

66. Branching in the A ray: same as B-E rays (0); different from B-E rays (1).

67. Highest position of an axillary on B-E ray primibrachials: IPr1 (0); IPr2 (1); ≥ IPr3 (2).

68. Bifurcation pattern on B-E ray primaxils: atomous (0); similar spacing (1); irregular spacing (2); plates fuse to form a mesh/fan shape (3).

69. Spacing between axillaries at and above the second brachitaxes in B-E rays: regular (0); irregular (1).

70. Secundaxil branching in the B-E rays: absent (0); present (1).

71. Branching above the secundaxil in B-E rays: absent (0); present (1).

72. Largest number of axillaries per ray: zero (0); one (1); two (2); ≥ 3 (3).

73. Outline pattern of bifurcation (Ubaghs, 1978, fig. 115): isotomous (0); heterotomous (# 2 and 3 in Ubaghs, 1978, fig 115) (1); endotomous (2); exotomous (3).

74. Pinnulation: absent (0); present (1).

75. Branching complexity of minor arm appendages on brachials distributed along the main ray axis: absent (0); ramulate (i.e., terminal appendages are separated by at least one brachial, branched or unbranched) (1); pinnulate (i.e., unbranched terminal appendages distributed along successive brachial plates) (2).

76. Mature (i.e., distal) brachial type: uniserial (0); biserial (1).

77. Transition from proximal uniserial to distal biseral brachials: absent (0); present (1).

78. Mature shape of uniserial brachials (sensu Webster 2007): rectilinear (0); weakly cuneate (1); moderately cuneate (2); strongly cuneate (3).

79. Distal biserial brachial shape (sensu Webster 2007): wedge (0); round (1); flat chisel (2).

80. Size of the primibrachials: smaller than radials (0); subequal to radials (1).

81. Number of axillaries in the A ray: same as other rays (0); first branching is higher than other rays (1).

82. Shape of the first primibrachial: tetragonal (0); pentagonal (1); triangular (2).

83. Dimensions of the first primibrachial: width > height (0); width ~ height (1); width < height (2).

84. Primaxil ornamentation: absent (0); present (1).

85. Maximum number of secundibrachials: zero (0); one (1); two (2); ≥ 3 (3).

86. Secundaxil spines: absent (0); present (1).

87. Syzygial sutures on brachials: absent (0); present (1).

88. Column: absent (0); present (1).

89. Proximal shape of the stalk: circular (0); pentagonal (1).

90. Lumen shape in proxistele columnals: circular (0); pentalobate (1); pentastellate (2).

91. Branching appendages on proximal stem: absent (0); present (1).

92. Stem appendages: absent (0); cirri (1); highly cirriferous (2).

Character Data and MrBayes commands

```
#NEXUS
BEGIN DATA;
	DIMENSIONS  NTAX=83 NCHAR=92;
	FORMAT DATATYPE=standard MISSING=?;
MATRIX
Adinocrinus	3	1	1	1	1	4	2	1	2	0
	?	1	2	2	1	2	1	1	1	2	1
	0	0	2	0	1	0	1	2	1	1	1
	1	1	1	0	0	1	0	1	0	2	0
	1	1	0	2	0	1	1	?	0	?	?
	?	?	?	?	?	?	?	0	0	0	1
	1	0	1	0	1	0	2	0	1	?	0
	0	0	?	2	1	1	1	0	3	0	0
	1	0	?	?	?
Aesiocrinus	3	1	1	1	0	?	1	1	1	0
	?	1	1	1	0	0	1	1	1	1	1
	0	0	2	0	0	0	?	2	1	1	1
	1	1	1	0	0	2	0	1	0	2	2
	1	1	0	2	0	1	1	1	0	0	0
	1	2	1	1	1	0	?	0	1	0	1
	0	1	1	?	0	0	1	0	1	2	0
	0	1	?	0	0	0	0	0	0	0	1
	1	1	?	1	2
Agassizocrinus		1	2	0	1	0	?	0	0	0
	2	?	1	0	2	0	0	1	1	0	0
	0	0	0	2	0	1	0	0	2	1	1
	1	1	0	1	0	0	1	0	1	0	0
	0	1	1	0	0	0	0	?	?	?	?
	?	?	?	?	?	?	?	?	0	1	0
	1	0	0	1	?	0	0	1	0	1	2
	0	0	1	?	0	0	1	0	0	0	0
	0	0	?	?	0	0
Amabilicrinus 2	0	0	0	0	?	0	0	0	0
	1	0	0	1	0	0	1	1	0	0	1
	0	0	2	0	?	0	0	2	1	?	?
	?	0	1	0	0	1	0	1	2	1	0
	1	1	2	1	0	0	1	1	?	0	1
	1	1	0	0	1	0	?	0	0	0	1
	1	1	1	1	1	1	3	2	1	2	0
	0	1	?	1	1	0	0	0	3	0	0
	1	0	1	0	0
Ampelocrinus 3	1	1	1	0	?	0	1	1	0
	1	0	0	1	0	0	1	1	0	0	1
	0	0	2	0	1	0	2	2	1	1	1
```

1	1	1	0	0	2	0	1	1	2	2
1	1	1	2	2	1	1	1	0	0	0
0	?	3	0	0	0	?	0	1	0	1
0	1	1	?	0	0	1	0	1	2	0
0	2	?	0	0	0	0	0	0	0	0
1	1	1	1	2						
Anobasicrinus	3	1	0	1	0	?	1	1	1	0
0	1	0	1	0	0	1	1	0	0	1
0	0	1	?	1	0	0	2	1	1	1
1	1	1	0	0	1	0	1	2	1	0
1	1	2	1	0	0	1	1	0	2	0
1	2	1	2	0	0	?	0	1	0	1
0	0	1	0	1	1	3	2	1	2	0
0	0	?	0	0	1	0	0	3	0	0
1	0	?	?	?						
Aphelecrinus	2	0	0	0	0	?	0	0	0	0
0	0	0	1	0	0	1	1	0	0	1
0	0	2	0	?	0	0	2	1	1	?
?	1	1	0	0	1	0	1	2	1	0
1	1	2	1	0	0	1	1	0	0	0
0	?	0	0	0	0	?	0	0	0	1
0	0	1	0	1	0	2	0	1	2	0
0	2	?	1	0	1	1	0	3	0	0
1	0	0	?	?						
Apographiocrinus		3	1	1	1	0	?	2	1	2
0	?	1	1	1	1	2	1	1	0	2
1	0	0	1	2	1	0	2	2	1	1
1	1	1	1	0	2	2	0	1	?	2
2	1	1	?	2	0	1	1	0	0	1
0	0	?	0	0	0	0	?	0	1	0
1	0	0	1	0	0	0	1	0	1	2
0	0	1	?	2	0	1	2	0	0	0
0	1	0	?	0	0					
Arkacrinus	3	1	1	1	0	?	2	1	2	0
2	1	2	2	0	2	1	1	1	2	1
0	0	2	1	1	0	1	2	1	1	1
1	1	1	1	?	?	2	0	?	?	?
?	0	?	?	?	?	?	?	?	?	?
?	?	?	?	?	?	?	0	1	0	1
?	?	?	?	?	?	?	?	1	2	?
?	?	?	?	?	?	?	?	?	?	?
1	0	?	?	?						
Barycrinus	2	1	0	1	1	3	0	0	0	0
1	0	0	2	0	2	1	1	0	1	1
0	0	0	?	1	1	1	1	0	0	0
0	1	1	0	1	1	0	1	1	0	0

0	0	?	?	?	?	1	?	?	0	0
0	?	0	0	0	0	?	0	0	0	1
0	1	1	?	0	0	1	1	0	1	0
0	0	?	0	0	0	0	0	3	0	0
1	0	0	0	0						
Basleocrinus	2	1	0	1	0	?	0	0	0	2
?	?	0	2	0	0	1	1	0	0	0
0	0	2	0	1	0	0	2	1	1	1
1	1	1	0	2	2	0	?	?	?	?
?	?	?	?	?	?	?	?	?	?	?
?	?	?	?	?	?	?	0	1	0	?
?	?	?	?	?	?	?	?	?	?	?
?	?	?	?	?	?	?	?	?	?	?
1	0	?	?	?						
Botryocrinus	2	0	0	0	0	?	0	0	0	0
1	0	0	1	0	0	1	0	0	0	1
0	0	1	?	0	1	1	0	0	?	?
?	1	1	0	1	1	0	1	0	0	0
0	0	?	?	?	?	1	1	?	0	0
1	1	3	0	0	0	?	0	0	0	1
0	1	1	1	1	0	2	1	0	1	0
0	0	?	0	0	0	0	0	2	0	0
1	1	1	0	0						
Blothrocrinus	2	0	0	0	0	?	0	0	0	0
1	0	0	2	0	0	1	1	0	0	0
0	0	2	0	1	0	0	2	1	1	1
1	1	1	0	0	1	0	1	2	1	0
1	1	2	1	0	1	1	1	0	0	0
0	?	0	0	0	0	?	0	0	0	1
0	1	1	1	1	1	3	1	1	2	0
0	2	?	0	0	0	0	0	3	0	0
1	0	?	0	0						
Bridgerocrinus	1	0	0	0	0	?	0	0	0	0
2	0	0	2	0	0	1	0	0	0	1
0	0	2	0	?	0	0	2	1	?	?
?	1	1	0	0	1	0	1	0	0	0
1	1	?	1	0	0	1	1	0	1	0
0	?	?	0	0	1	0	0	1	0	1
0	1	1	0	0	0	1	0	1	2	0
0	1	?	2	0	0	2	0	0	0	0
1	0	?	?	?						
Bursacrinus	3	0	0	1	1	4	0	0	0	0
0	0	0	2	1	0	1	1	0	0	0
0	0	2	0	1	0	0	2	1	1	1
1	1	1	0	2	2	0	?	?	?	?
?	?	?	?	?	?	1	?	?	?	?

?	?	?	?	?	?	?	0	0	0	1
0	0	1	0	1	0	2	0	1	2	0
0	1	?	0	0	0	0	0	3	0	0
1	?	?	?	?						
Cadocrinus	2	1	0	1	0	?	2	1	2	0
?	0	1	2	0	2	1	1	0	2	1
0	0	2	1	1	0	2	2	1	1	1
1	1	1	0	1	2	0	0	?	?	?
?	0	?	?	?	?	1	1	0	1	0
1	2	1	2	0	0	?	0	1	0	?
?	?	?	?	?	?	?	?	1	2	?
?	?	?	?	?	?	?	?	?	?	?
1	0	?	?	?						
Calceolispongia		3	1	1	1	0	?	2	1	1
0	2	1	1	2	0	2	1	1	0	0
0	0	0	2	0	1	0	?	2	1	1
1	1	1	1	0	0	2	0	1	?	2
2	1	1	?	2	0	1	0	0	1	?
?	?	?	?	?	0	0	?	0	1	0
0	0	?	0	?	0	0	0	?	1	2
0	0	3	?	0	0	0	0	0	3	1
1	1	1	?	1	2					
Delocrinus	3	1	1	1	0	?	2	1	2	0
?	1	1	1	0	2	1	1	0	1	0
0	0	2	0	1	0	0	2	1	1	1
1	1	1	0	2	2	0	1	?	2	?
?	1	?	2	?	?	1	1	0	0	0
0	?	1	1	0	1	0	0	1	0	1
0	0	1	?	0	0	1	0	1	2	1
1	?	1	2	0	2	2	0	3	0	0
1	0	?	?	?						
Cercidocrinus	2	0	0	0	0	?	0	0	0	0
0	0	0	1	0	0	1	1	0	0	1
0	0	2	0	?	0	0	2	1	?	?
1	1	1	0	0	1	0	1	2	0	0
1	1	2	1	0	0	1	1	?	?	?
1	?	3	2	0	0	?	0	1	0	1
1	0	1	0	1	1	3	2	1	2	0
0	0	?	2	1	1	1	0	3	0	0
1	0	?	?	?						
Clathrocrinus	3	1	0	1	0	?	2	1	2	0
?	1	1	1	0	1	1	1	0	0	1
0	0	2	0	1	0	0	2	1	1	1
1	1	1	0	0	1	0	1	0	0	1
0	1	0	1	2	2	1	2	0	0	0
0	?	3	0	1	1	1	0	0	0	1

0	0	1	?	0	0	1	0	1	2	0
0	1	?	1	0	2	2	0	0	0	0
1	0	?	0	0						
Codiacrinus	2	2	0	0	1	0	0	0	0	1
0	0	0	2	0	1	0	0	0	0	0
0	0	0	?	2	1	1	0	0	1	0
0	1	0	?	?	?	?	0	?	?	?
?	0	?	?	?	?	0	0	?	?	0
0	?	?	?	0	0	?	0	0	0	1
0	2	?	?	1	1	3	0	0	0	0
0	0	0	0	0	0	?	0	3	0	0
1	0	?	?	?						
Corematocrinus		2	0	0	0	1	0	0	0	0
0	0	0	0	2	0	0	1	1	0	0
1	0	0	0	?	2	1	0	?	?	?
?	?	1	1	0	2	1	0	1	0	1
1	1	1	2	1	0	0	1	1	0	0
0	1	0	3	?	0	0	?	0	1	0
1	0	1	1	?	0	0	1	0	1	2
0	0	1	?	1	0	0	1	0	0	0
0	1	1	?	0	0					
Corythocrinus	2	0	0	0	0	?	0	0	0	0
2	0	0	2	0	0	1	1	0	0	0
0	0	2	0	1	0	0	2	1	1	1
1	1	1	0	0	2	0	0	?	?	?
?	0	?	?	?	?	1	?	?	0	0
0	?	0	?	0	0	?	0	1	0	1
0	3	1	0	1	1	3	0	1	2	0
0	3	?	0	0	0	0	0	3	0	1
1	0	?	0	0						
Cricocrinus	3	1	0	1	0	?	1	0	2	0
0	0	2	2	0	0	0	1	0	1	1
0	0	2	0	1	0	0	2	1	1	1
1	1	1	1	?	2	2	0	?	?	?
?	0	?	?	?	?	1	?	?	?	?
?	?	?	?	?	?	?	0	1	0	1
0	0	1	?	0	0	1	0	1	2	1
1	?	2	0	0	2	?	0	0	0	0
1	0	?	?	?						
Cromyocrinus	3	2	0	0	0	?	0	0	0	0
?	1	0	1	0	1	1	1	0	1	0
0	0	2	2	1	0	0	2	1	1	1
1	1	1	0	0	1	0	1	2	1	0
1	1	0	1	0	1	1	0	0	0	0
0	?	0	1	0	0	?	0	0	0	0
?	?	0	?	0	0	0	?	1	2	0

0 1 ? 0 0 0 0 0 0 0 0
1 0 ? 0 0
Crotalocrinites 2 2 0 1 1 0 0 0 0 0
0 0 0 0 0 1 1 1 0 1 0
0 0 0 ? 1 ? 0 ? 0 ? 0
? 1 1 0 0 2 0 0 ? ? ?
? 0 ? ? ? ? 0 0 ? ? 0
0 ? ? ? 0 0 ? 0 1 1 1
0 1 3 0 1 1 2 2 0 0 0
0 0 ? 0 0 2 0 0 3 0 0
1 0 0 0 0
Cupressocrinities 3 1 1 1 0 ? 1 1 1
2 ? ? 1 1 0 1 0 1 0 1
1 0 0 2 0 1 0 0 3 1 1
0 0 1 0 ? ? ? ? 0 ? ?
? ? 0 ? ? ? ? 1 0 ? ?
0 0 ? ? ? 0 0 ? 0 1 0
0 0 ? 0 ? 0 0 0 ? 1 3
0 0 0 ? 0 0 0 0 0 0 0
0 1 ? ? 1 1
Cupulocrinus 2 0 0 0 0 ? 0 0 0 0
1 0 0 1 1 0 1 1 0 0 0
0 0 1 ? 1 0 0 1 1 1 1
0 1 1 0 0 0 0 1 2 1 0
0 0 ? ? ? ? 1 1 0 0 1
0 ? 0 0 0 0 ? 0 0 0 1
0 2 1 0 1 1 3 0 0 0 0
0 0 ? 0 0 0 0 0 3 0 0
1 0 0 0 0
Cyathocrinites 2 2 0 0 1 0 0 0 0 0
0 0 0 1 0 1 1 1 0 0 0
0 0 0 ? 2 1 1 1 1 0 0
0 1 1 0 1 2 0 0 ? ? ?
? 0 ? ? ? ? 1 1 0 0 0
0 ? 1 0 0 0 ? 0 0 0 1
0 2 1 1 1 1 3 0 0 0 0
0 0 ? 0 0 0 0 0 3 0 0
1 0 0 0 0
Cymbiocrinus 3 1 0 1 0 ? 2 1 2 0
? 1 1 ? 0 2 1 1 0 0 1
0 0 2 0 1 0 0 2 1 1 1
1 1 1 0 0 2 0 1 0 2 2
1 1 0 2 ? 1 1 0 0 0 0
0 ? 0 ? 0 0 ? 0 1 0 1
0 1 1 ? 0 0 1 0 1 2 0

0	3	?	0	0	0	0	0	0	0	1
1	1	?	1	2						
Decadocrinus	3	1	0	1	0	?	2	1	2	0
0	1	1	1	0	2	1	1	0	0	0
0	0	2	0	1	0	1	2	1	1	1
1	1	1	0	0	1	0	1	0	1	0
1	1	0	1	0	1	1	1	0	0	0
0	?	0	2	0	0	?	0	1	0	1
1	1	1	?	0	0	1	0	1	2	0
0	3	?	0	1	0	0	0	0	0	0
1	1	?	0	0						
Dendrocrinus	2	0	0	0	0	?	0	0	0	0
2	0	0	2	1	0	1	1	0	0	1
0	0	0	?	0	1	1	0	0	?	?
?	1	1	0	0	0	0	1	2	0	0
0	1	2	2	1	1	1	1	0	0	0
1	1	3	0	0	0	?	0	0	0	1
1	2	2	0	1	1	3	0	0	0	0
0	0	?	0	0	0	0	0	3	0	0
1	0	?	0	0						
Diphuicrinus	3	1	0	1	1	4	2	1	2	0
?	1	1	1	0	2	1	1	0	1	?
0	0	2	1	1	0	0	2	1	1	1
1	1	1	0	0	2	0	1	?	2	2
1	1	?	2	?	?	1	?	?	?	?
?	?	?	?	?	?	?	0	1	0	1
0	0	1	?	0	0	1	0	1	2	0
0	3	?	0	0	1	0	1	0	0	0
1	0	?	0	0						
Erisocrinus	2	0	1	0	0	?	1	0	1	0
0	0	0	1	0	1	0	1	0	1	1
0	0	2	0	1	0	0	2	1	1	1
1	1	1	1	?	?	2	0	?	?	?
?	0	?	?	?	?	1	1	0	0	0
0	?	0	0	0	0	?	0	1	0	1
0	0	1	?	0	0	1	0	1	2	1
1	?	2	2	0	1	0	0	0	0	0
1	0	0	0	0						
Eupachycrinus	3	1	0	1	0	?	2	1	2	0
2	1	1	2	0	2	1	1	0	1	0
0	0	2	1	1	0	0	2	1	1	1
1	1	1	0	0	1	0	1	0	0	0
1	1	0	1	0	0	1	0	?	?	?
?	?	?	?	0	1	0	0	1	0	1
1	0	1	0	1	0	2	0	1	2	1

1	?	2	0	1	1	0	0	3	0	0
1	0	?	0	0						
Euspirocrinus	2	1	0	0	0	?	0	0	0	0
0	0	0	1	0	0	1	1	0	0	1
0	0	0	?	2	1	1	0	0	0	0
0	1	1	0	0	1	0	1	2	0	0
1	1	2	0	0	0	1	1	0	1	0
0	?	0	1	0	?	?	0	0	0	1
0	1	1	?	1	1	3	0	0	0	0
0	0	?	0	0	0	0	0	3	0	0
1	0	?	0	0						
Exocrinus	2	1	0	?	0	?	1	1	2	0
?	1	0	1	0	1	1	1	0	0	1
0	0	2	0	1	0	0	2	1	1	1
1	1	1	0	2	2	0	1	0	2	1
1	1	0	2	?	1	1	0	?	0	0
0	?	0	0	0	0	?	0	1	0	1
0	0	1	1	1	1	3	1	1	3	0
0	0	?	2	0	1	2	0	3	0	0
1	0	?	0	0						
Galateacrinus	3	1	0	1	1	4	2	1	2	0
?	1	2	1	0	2	1	1	1	2	1
0	0	2	2	1	0	0	2	1	1	1
1	1	1	0	?	1	0	1	?	0	0
1	1	?	0	0	1	1	?	?	?	?
?	?	?	?	?	?	?	0	1	0	1
0	0	1	1	1	1	3	2	1	2	0
0	3	?	0	0	1	?	0	3	0	0
1	0	?	0	0						
Gasterocoma	1	2	0	0	0	?	0	0	1	2
?	1	0	2	0	1	1	2	0	1	1
0	0	0	?	2	1	1	0	0	1	0
0	1	1	0	1	2	0	0	?	?	?
?	0	?	?	?	?	0	0	?	?	0
0	?	?	?	0	0	?	0	0	0	1
1	?	?	0	1	1	3	0	0	0	0
0	0	?	0	0	0	0	0	3	0	0
1	0	?	0	0						
Glossocrinus	2	0	0	0	1	1	0	0	0	0
0	0	0	1	0	0	1	0	0	0	1
0	0	1	?	1	1	0	0	0	?	1
?	1	1	0	0	1	0	1	0	1	0
1	1	2	1	0	0	1	1	?	0	1
1	1	3	0	0	0	?	0	0	0	1
0	2	1	?	1	0	2	0	1	2	0

0	1	?	0	0	0	2	0	3	0	0
1	1	?	1	1						
Graphiocrinus	4	1	0	1	0	?	2	1	2	0
?	1	2	0	0	2	1	1	1	2	1
0	0	2	0	1	0	1	2	1	1	1
1	1	1	0	2	2	0	0	?	?	?
?	0	?	?	?	?	1	?	?	?	?
?	?	?	?	?	?	?	0	1	0	1
0	0	1	?	0	0	1	0	1	2	0
0	0	?	2	0	1	2	0	0	0	0
1	0	?	0	0						
Hydreionocrinus		3	1	0	1	0	?	0	0	0
0	?	0	0	1	0	?	1	1	0	0
1	0	0	2	0	1	0	1	2	1	1
1	1	1	1	0	0	1	0	1	0	1
0	1	1	0	1	0	0	1	2	0	?
0	?	?	?	2	1	1	2	0	1	0
1	0	0	1	1	1	1	3	3	1	2
1	?	?	1	1	0	1	0	0	3	0
0	1	0	?	0	0					
Indocrinus	1	0	1	1	1	3	0	0	0	1
2	0	0	2	0	2	1	1	0	0	0
0	0	1	?	0	1	1	?	?	?	?
?	?	1	0	1	2	0	0	?	?	?
?	0	?	?	?	?	0	0	0	?	?
?	?	?	?	?	?	?	1	1	0	?
?	?	?	?	?	?	?	?	?	?	?
?	?	?	?	?	?	?	?	?	?	0
1	?	?	?	?						
Laudonocrinus	3	1	0	1	0	?	1	0	2	0
0	1	1	1	0	2	1	1	1	2	1
0	0	1	2	1	0	1	2	1	1	1
1	1	1	0	?	1	0	1	?	1	0
1	1	?	2	0	0	1	?	?	0	0
1	3	?	2	1	1	2	0	1	0	1
0	0	1	0	1	1	?	2	1	2	0
0	0	?	0	0	1	0	1	?	0	0
1	0	?	?	?						
Lecythocrinus	3	0	0	0	0	?	0	0	0	2
?	0	0	2	0	0	1	0	0	0	0
0	0	0	?	?	1	1	0	0	0	0
0	1	1	0	2	2	0	1	2	0	0
1	0	?	?	?	?	1	1	0	0	0
0	?	0	0	0	0	?	0	0	0	1
0	2	1	0	1	1	3	0	0	0	0

0	0	?	0	0	0	0	0	3	0	0
1	1	?	0	0						
Lophocrinus	2	0	0	0	0	?	0	0	0	0
2	0	0	2	0	0	1	0	0	0	0
0	0	1	?	?	?	0	?	?	?	?
?	1	1	0	0	2	0	1	?	2	2
1	1	?	2	?	1	1	1	0	0	0
0	?	1	0	0	0	?	0	1	0	0
0	?	0	?	0	0	0	?	0	1	0
0	3	?	0	0	0	0	0	0	0	0
1	0	0	0	0						
Manicrinus	2	0	0	0	0	?	0	0	0	1
1	0	0	2	1	0	1	1	0	0	0
1	1	0	?	0	1	1	0	0	0	0
0	1	1	0	0	2	0	0	?	?	?
?	0	?	?	?	?	0	0	?	?	0
0	?	?	?	0	0	?	0	0	0	1
0	?	?	?	?	?	?	?	0	0	0
0	0	?	0	0	0	0	0	?	0	0
1	0	?	?	?						
Mastigocrinus	2	0	0	0	0	?	0	0	0	0
2	0	0	2	0	0	1	0	0	0	1
0	0	1	?	0	1	1	0	0	?	1
0	1	1	0	1	2	0	0	?	?	?
?	0	?	?	?	?	1	1	0	0	0
1	1	3	0	0	0	?	0	0	0	1
0	2	2	1	1	1	2	1	0	0	0
0	0	?	0	0	0	0	0	3	0	0
1	0	?	0	0						
Mollocrinus	2	2	0	1	0	?	0	0	0	1
?	0	0	1	0	1	1	1	0	0	0
0	0	0	?	0	1	1	1	1	0	1
?	1	1	0	1	1	0	1	0	0	0
0	0	?	?	?	?	?	?	?	?	?
?	?	?	?	?	?	?	0	0	0	?
?	?	?	?	?	?	?	?	?	?	?
?	?	?	?	?	?	?	?	?	?	?
1	?	?	?	?						
Oligobrachyocrinus		1	0	0	0	1	4	0	0	0
0	2	0	0	2	0	0	?	2	0	0
0	0	?	0	?	0	1	1	1	1	?
?	?	1	?	?	?	?	?	0	?	?
?	?	0	?	?	?	?	0	0	1	?
?	?	?	?	?	0	0	?	1	0	0
?	?	?	?	?	?	?	?	?	0	?

0	0	?	?	?	?	?	?	?	?	0
0	1	0	?	?	?					
Ottawacrinus	1	0	0	0	0	0	0	0	0	0
1	0	0	1	1	0	1	0	0	0	0
1	0	1	?	?	0	0	?	?	?	?
?	1	1	0	2	0	0	1	2	0	0
0	1	2	1	1	2	1	1	0	0	0
0	?	0	0	0	0	?	0	1	1	1
0	2	2	1	1	1	3	1	0	0	0
0	0	?	1	0	0	1	0	3	0	0
1	0	1	0	0						
Pachylocrinus	3	1	0	1	0	?	1	1	2	0
?	0	0	1	0	1	1	1	0	0	0
0	0	1	?	1	0	0	2	1	1	1
1	1	1	0	0	1	0	1	2	1	0
1	1	2	1	0	0	1	1	0	0	0
?	?	?	0	0	0	?	0	0	0	1
0	1	1	1	1	1	3	1	1	2	0
0	2	?	0	0	0	0	0	3	0	0
1	0	?	0	0						
Paradelocrinus	3	1	0	1	0	?	2	1	2	0
?	1	2	2	0	2	0	1	1	2	1
0	0	2	1	1	0	1	2	1	1	1
1	1	1	1	?	2	2	0	?	?	?
?	0	?	?	?	?	?	?	?	?	?
?	?	?	?	?	?	?	0	1	0	?
?	?	?	?	?	?	?	?	?	?	?
?	?	?	?	?	?	?	?	?	?	?
1	0	?	?	?						
Pelecocrinus	2	1	0	1	0	?	0	0	0	0
0	0	0	1	0	0	1	1	0	0	1
0	0	1	?	1	1	1	?	1	1	1
?	1	1	0	0	1	0	1	0	0	0
1	1	2	1	0	0	1	0	1	1	0
0	?	?	2	0	1	1	0	0	0	1
1	2	1	1	1	1	3	1	1	2	0
0	3	?	0	1	1	0	0	3	0	0
1	1	?	0	0						
Petalocrinus	3	1	1	0	0	?	1	1	2	2
?	?	1	2	0	1	0	1	0	0	1
0	0	0	?	0	1	1	0	0	?	0
?	1	0	?	?	?	?	0	?	?	?
?	0	?	?	?	?	0	0	?	?	0
0	?	?	?	0	0	?	0	0	1	?
0	1	3	?	?	?	?	?	0	0	?

?	?	?	0	?	2	0	0	?	0	0
1	0	?	?	?						
Phanocrinus	3	1	0	1	0	?	2	2	2	0
?	1	1	1	0	1	1	1	1	0	0
0	0	2	0	1	0	0	2	1	1	1
?	1	1	0	0	1	0	1	2	1	0
1	1	2	1	0	0	1	0	1	?	0
?	?	?	?	?	1	0	0	1	0	1
0	0	1	?	0	0	1	0	1	2	0
?	1	?	0	0	1	0	0	0	0	0
1	0	1	0	0						
Pirasocrinus	4	1	1	1	0	?	2	1	2	0
?	1	2	2	1	2	1	1	1	2	1
0	0	2	0	1	0	1	2	1	1	1
1	1	1	0	0	1	0	1	2	1	0
1	1	0	2	0	1	1	2	1	1	0
0	?	?	2	?	1	2	0	1	0	0
0	0	1	0	1	1	3	0	1	2	0
0	0	?	2	0	1	0	1	3	1	0
1	0	?	0	0						
Plicodendrocrinus		2	0	0	0	1	1	0	0	0
0	0	0	0	2	0	0	1	1	0	0
1	0	0	0	?	0	1	1	0	0	?
?	?	1	1	0	0	0	0	1	2	0
0	0	1	2	2	1	1	1	1	0	0
0	1	1	3	0	0	0	?	0	0	0
1	1	2	2	0	1	1	3	0	0	0
0	0	0	?	0	0	0	0	0	3	0
0	1	1	?	0	0					
Poteriocrinites	2	0	0	0	0	?	0	0	0	0
1	0	0	1	0	0	1	0	0	0	1
0	1	0	?	0	1	1	1	1	0	0
?	1	1	0	0	1	0	1	2	0	0
1	1	2	0	0	0	1	1	0	0	0
1	1	3	0	0	0	?	0	0	0	1
0	2	1	0	1	1	3	0	1	2	0
0	0	?	0	0	0	0	0	3	0	0
1	0	1	1	1						
Proctothylacocrinus		2	0	0	0	1	0	0	0	0
0	0	0	0	2	0	0	1	1	0	0
0	0	0	1	?	1	0	0	0	0	?
?	?	1	1	0	0	1	0	1	2	0
0	1	1	2	2	0	0	1	1	0	0
1	1	3	2	0	0	0	?	0	0	0
1	1	2	1	1	1	1	3	0	0	0

0	0	0	?	0	0	0	0	0	3	0
0	1	0	?	0	0					
Protencrinus	3	1	1	1	0	?	2	1	2	0
?	1	2	2	0	2	1	1	1	2	1
0	0	2	0	1	0	0	2	1	1	1
1	1	1	1	?	2	2	0	?	?	?
?	0	?	?	?	?	1	?	?	?	?
?	?	?	?	?	?	?	0	1	0	1
0	0	1	?	0	0	1	0	1	2	1
1	?	1	1	0	1	0	0	0	0	0
1	?	?	?	?						
Rhenocrinus	2	0	0	0	0	?	0	0	0	0
0	0	0	0	0	0	1	1	0	0	?
0	0	1	?	1	0	0	?	?	?	?
?	1	1	0	2	1	0	1	?	?	0
1	1	?	?	0	0	1	1	0	0	0
0	?	0	1	0	0	?	0	0	0	1
?	2	2	?	0	0	1	0	0	1	0
0	0	?	1	?	0	0	0	0	0	0
1	0	?	0	0						
Rutkowskicrinus		3	0	0	0	1	1	0	0	0
0	0	0	0	1	1	0	1	1	0	0
1	0	0	1	?	1	0	1	1	1	1
0	0	1	1	0	0	2	0	0	?	?
?	?	0	?	?	?	?	1	1	0	0
1	1	1	?	0	0	0	?	0	0	0
1	0	2	1	0	0	0	2	1	1	2
0	0	0	?	0	0	0	0	0	3	0
0	1	2	?	1	1					
Scotiacrinus	3	1	0	1	1	4	1	1	2	0
?	1	1	1	0	1	1	1	0	1	1
0	0	2	0	1	0	0	2	1	1	1
?	1	1	0	0	1	0	1	2	1	0
1	1	?	1	0	0	1	?	?	?	?
?	?	?	?	?	?	?	0	1	0	1
0	0	1	?	0	0	1	1	1	2	0
0	1	?	0	0	1	0	0	0	0	0
1	0	?	?	?						
Sellardsicrinus	3	1	0	1	0	?	1	1	2	0
0	1	1	1	0	2	1	1	0	1	1
0	0	2	0	1	?	0	2	1	1	1
?	1	1	0	?	1	0	1	?	1	0
1	1	?	1	0	0	1	?	?	?	?
?	?	?	?	?	?	?	0	1	0	1
0	0	1	0	1	1	3	0	1	2	1

0	?	0	0	0	1	0	0	3	0	0
1	0	?	?	?						
Sostronocrinus		2	0	0	0	1	1	0	0	0
0	0	0	0	1	0	0	1	1	0	0
1	0	0	1	?	0	0	0	2	1	1
1	1	1	1	0	0	1	0	1	?	1
0	1	1	?	1	0	0	1	1	0	0
1	1	0	0	0	0	0	?	0	1	0
1	0	1	1	0	1	0	2	0	1	2
0	0	2	?	0	0	0	0	0	3	0
0	1	1	?	?	?					
Scytalocrinus	3	1	0	1	0	?	1	0	1	0
2	1	0	0	0	0	1	1	0	0	1
0	0	2	0	1	0	0	2	1	1	1
1	1	1	0	2	1	0	1	0	1	1
1	1	2	1	1	0	1	1	0	0	0
0	?	0	0	0	0	?	0	1	0	1
0	0	1	?	0	0	1	0	1	2	0
0	1	?	1	0	1	1	0	0	0	0
1	0	?	0	0						
Spaniocrinus	3	1	1	1	0	?	0	0	0	0
0	0	0	0	0	0	0	1	0	0	1
0	0	2	0	1	0	0	2	1	1	1
?	1	0	?	?	?	?	0	?	?	?
?	0	?	?	?	?	?	?	?	?	?
?	?	?	?	?	?	?	0	1	1	0
?	?	0	?	0	0	0	?	1	2	0
0	0	?	2	?	0	0	0	0	0	0
1	0	?	?	?						
Sphaerocrinus	1	2	0	1	1	0	0	0	0	0
0	0	0	1	0	1	1	0	0	0	0
0	0	0	?	1	1	1	0	0	0	0
0	1	1	0	?	1	0	1	?	0	0
0	0	?	?	?	?	0	0	1	?	?
0	?	?	?	0	0	?	0	0	0	?
0	?	?	?	?	?	?	?	0	0	0
0	0	?	?	?	0	?	0	?	0	0
1	?	?	?	?						
Stachyocrinus	3	1	1	1	0	?	2	1	2	0
?	1	2	2	0	2	0	1	1	2	1
0	0	2	0	1	0	0	2	1	1	1
?	1	1	1	?	2	2	0	?	?	?
?	0	?	?	?	?	1	?	?	?	?
?	?	?	?	?	?	?	0	1	1	1
0	0	1	?	0	0	1	0	1	2	0

0	0	?	2	0	1	2	0	0	0	0
1	0	?	?	?						
Staphylocrinus	2	1	0	1	0	?	1	1	2	2
?	1	0	1	0	2	1	1	0	2	0
0	0	2	0	1	0	0	2	1	1	1
1	1	1	0	0	1	0	1	0	1	0
1	1	?	1	0	0	?	?	?	?	?
?	?	?	?	?	?	?	0	1	0	1
0	0	1	0	1	?	?	0	1	2	0
0	1	?	0	0	1	0	0	3	0	0
0	0	?	0	0						
Stellarocrinus	3	1	1	1	1	1	2	1	2	0
0	1	1	1	0	2	1	1	0	1	1
0	0	2	0	1	1	1	2	1	?	1
?	1	1	0	0	2	0	1	0	2	1
1	1	0	2	0	1	1	2	0	0	0
1	1	2	0	1	1	1	0	1	0	1
0	0	1	0	1	1	3	0	1	2	1
0	?	1	0	0	1	0	0	3	0	0
1	0	?	0	0						
Streblocrinus	1	2	0	0	0	?	0	0	0	0
2	0	0	2	1	0	1	2	0	0	0
0	0	0	?	1	1	1	0	0	0	0
0	1	1	0	1	1	0	0	?	?	?
?	0	?	?	?	?	0	0	0	?	0
0	?	?	?	0	0	?	1	0	0	?
?	?	?	?	?	?	?	?	0	0	0
0	0	?	0	?	0	?	0	?	0	0
1	?	?	?	?						
Sundacrinus	2	1	0	1	1	4	0	0	1	0
0	0	0	1	1	1	1	1	0	0	0
0	0	2	0	1	0	2	2	1	1	1
?	1	1	0	0	2	0	1	?	2	2
1	1	?	2	0	?	?	?	?	?	?
?	?	?	?	?	?	?	1	1	0	?
?	?	?	?	?	?	?	?	?	?	?
?	?	?	?	?	?	?	?	?	?	?
1	0	?	?	?						
Sycocrinites	0	2	0	0	0	?	0	0	0	1
2	0	0	2	0	0	1	0	0	0	0
0	0	0	?	0	1	?	0	0	0	0
0	1	0	?	?	?	?	0	?	?	?
?	0	?	?	?	?	0	0	1	?	0
0	?	?	?	0	0	?	2	?	0	0
?	?	0	?	0	0	0	?	0	0	0

0	0	?	?	?	?	?	?	0	0	0
1	0	?	?	?						
Texacrinus	3	1	0	1	0	?	2	1	2	0
?	1	1	1	0	2	1	1	0	1	1
0	0	2	0	1	0	0	2	1	1	?
?	1	1	0	0	2	0	1	0	1	1
1	1	0	1	0	1	1	?	0	0	0
0	1	3	0	?	0	?	0	1	0	1
0	0	1	1	1	1	3	3	1	2	0
0	1	?	1	0	1	1	0	3	0	0
1	0	?	?	?						
Thalamocrinus	1	2	0	1	0	?	0	0	0	0
2	0	0	2	0	0	1	1	0	0	0
0	0	1	?	1	1	0	0	0	0	0
0	1	1	0	2	1	0	1	0	0	0
0	0	?	?	?	?	1	1	0	0	0
0	?	0	0	0	0	?	0	0	0	1
0	?	?	?	1	?	?	0	0	0	0
0	0	?	0	?	0	?	0	?	0	0
1	0	?	?	?						
Thenarocrinus	2	0	0	0	0	?	0	0	0	0
0	0	0	1	0	0	1	1	0	0	0
0	0	1	?	1	0	1	?	?	?	?
?	1	1	0	0	1	1	1	2	0	0
0	1	0	1	2	0	1	1	0	0	1
1	1	3	1	0	0	?	0	0	1	1
1	2	1	1	1	1	2	0	0	0	0
0	0	?	0	?	0	0	0	3	0	0
1	0	?	0	0						
Timorechinus	3	1	1	1	0	?	2	1	2	2
?	1	1	1	1	2	0	1	0	1	1
0	0	2	0	1	0	0	2	1	1	?
?	1	1	0	0	2	2	0	?	?	?
?	0	?	?	?	?	1	0	1	1	0
1	1	?	?	0	0	?	0	1	0	1
0	0	1	?	0	0	1	1	1	2	0
0	0	?	2	0	1	2	0	0	0	0
1	0	?	?	?						
Tribrachyocrinus		2	1	0	1	0	?	1	1	1
1	?	1	0	2	1	1	1	0	0	1
0	0	0	2	0	1	0	0	2	1	1
1	1	1	1	0	0	1	0	1	1	0
0	1	1	?	1	0	0	1	0	0	?
?	?	?	?	?	?	?	?	1	1	0
1	0	1	1	0	1	?	?	?	1	2

0	0	2	?	0	0	0	0	0	3	0
1	1	0	?	0	0					
Trimerocrinus	1	2	0	0	0	?	0	0	0	1
1	0	0	1	1	1	1	0	0	0	0
0	0	2	0	1	0	2	2	1	1	1
1	1	1	0	0	0	0	1	1	0	0
0	0	?	?	?	?	?	?	?	?	?
?	?	?	?	?	?	?	0	1	0	?
?	?	?	?	?	?	?	?	?	?	?
?	?	?	?	?	?	?	?	?	?	?
1	0	?	?	?						
Ulocrinus	1	2	0	0	0	?	0	0	0	0
2	0	0	2	0	0	1	1	0	0	0
0	0	2	0	1	0	0	2	1	1	1
1	1	1	0	0	1	0	1	1	0	1
0	0	?	?	?	?	1	?	?	?	?
?	?	?	?	?	?	?	0	1	0	1
0	0	1	?	0	0	1	0	1	2	1
1	?	1	0	0	1	0	0	0	0	0
1	0	1	0	0						
Zeacrinites	3	1	1	2	0	?	2	1	2	0
2	1	1	2	0	1	1	0	1	1	1
0	0	2	0	1	0	0	2	1	1	1
1	1	1	0	0	1	1	1	2	2	0
1	1	2	2	0	0	1	0	0	0	0
0	?	0	1	0	0	?	0	0	0	1
1	1	1	1	1	1	3	2	1	3	0
0	0	?	0	1	0	0	0	3	0	0
1	0	?	0	0						

;
END;

Begin mrbayes;
set autoclose=yes nowarn=yes;
outgroup Cupulocrinus;
[set up likelihood model of character evolution to MkV + gamma distributed rates]
lset rates=gamma Ngammacat=4 coding=variable;
[set up clade priors based on Wright (2017)]
constraint Root = 1-27 29-83;
constraint node1 = 45 47 36 55 26;
constraint node2 partial = 73 77 69 21 39 : 28 57 45 36 47 55 26 58 32 12 48 78 60 62 63 40 22 4;
constraint node3 partial = 62 63 40 22 4 : 1-3 5-14 16-21 23-39 41-61 64-65 67-83;

[Additional constraints]

constraint node4 partial = 1 57 2 5 17 30 31 3 14 81 82 6 7 8 9 53 24 34 61 70 79 11 13 15 16 18 19 20 23 25 27 68 33 35 37 38 41 42 44 46 52 54 56 64 65 67 71 72 74 76 80 83 4 22 62 40 63 66 : 21 39 43 50 75 73 69 29 49 77 12 48 32 58 26 55 47 45 36;

constraint node5 partial = [10] 21 39 43 50 75 73 69 29 49 77 12 48 32 58 26 55 47 45 36 78 60 59 : 1 57 2 5 17 30 31 3 14 81 82 6 7 8 9 53 24 34 61 70 79 11 13 15 16 18 19 20 23 25 27 68 33 35 37 38 41 42 44 46 52 54 56 64 65 67 71 72 74 76 80 83 4 22 62 40 63 66;

constraint node6 partial = [10] 21 39 43 50 75 73 69 29 49 77 12 48 32 58 26 55 47 45 36 [59] : 1 57 2 5 17 30 31 3 14 81 82 6 7 8 9 53 24 34 61 70 79 11 13 15 16 18 19 20 23 25 27 68 33 35 37 38 41 42 44 46 52 54 56 64 65 67 71 72 74 76 80 83 4 22 62 40 63 66 78 60;

constraint node7 partial = [10] 21 39 43 50 75 73 69 29 49 77 : 1 57 2 5 17 30 31 3 14 81 82 6 7 8 9 53 24 34 61 70 79 11 13 15 16 18 19 20 23 25 27 68 33 35 37 38 41 42 44 46 52 54 56 64 65 67 71 72 74 76 80 83 4 22 62 40 63 66 60 78;

constraint node8 partial = 69 21 39 73 75 50 43 : 1 57 2 5 17 30 31 3 14 81 82 6 7 8 9 53 24 34 61 70 79 11 13 15 16 18 19 20 23 25 27 68 33 35 37 38 41 42 44 46 52 54 56 64 65 67 71 72 74 76 80 83 4 22 62 40 63 66 12 32 48 58 59;

constraint node9 = 27 68;

constraint node10 partial = 2 5 17 30 : 1 3-4 6-16 18-21 23-29 32-83;

[calibrate priors on stratigraphic ranges] [fixed ages are midpoints of geologic stages of species FAD]

calibrate Adinocrinus = fixed(70.0);
calibrate Aesiocrinus = fixed(36.55); [Kasimovian]
calibrate Agassizocrinus = fixed(66.15);
calibrate Amabilicrinus = fixed(108.65);
calibrate Ampelocrinus = fixed(58.25);
calibrate Anobasicrinus = fixed(34.15);
calibrate Aphelecrinus = fixed(66.15);
calibrate Apographiocrinus = fixed(34.15);
calibrate Arkacrinus = fixed(50.40);
calibrate Barycrinus = fixed(70.0);
calibrate Botryocrinus = fixed(161.6);
calibrate Blothrocrinus = fixed(70.0);
calibrate Bridgerocrinus= fixed(84.0);
calibrate Bursacrinus = fixed(84.0);
calibrate Delocrinus = fixed(36.55);
calibrate Cercidocrinus = fixed(70.0);
calibrate Clathrocrinus = fixed(36.55);
calibrate Codiacrinus = fixed(121.7);
calibrate Corematocrinus = fixed(108.65);
calibrate Corythocrinus = fixed(70.0);
calibrate Cricocrinus = fixed(46.30);
calibrate Cromyocrinus = fixed(42.3);
calibrate Crotalocrinites= fixed(159.4);
calibrate Cupressocrinities = fixed(121.7);

```
calibrate	Cupulocrinus	= fixed(180.3);
calibrate	Cyathocrinites	=	fixed(84.0);
calibrate	Cymbiocrinus	=	fixed(58.25);
calibrate	Decadocrinus	=	fixed(70.0);
calibrate	Dendrocrinus	=	fixed(161.6);
calibrate	Diphuicrinus	=	fixed(50.40);
calibrate	Erisocrinus	= fixed(38.25);
calibrate	Eupachycrinus=	fixed(66.15);
calibrate	Euspirocrinus	=	fixed(161.6);
calibrate	Exocrinus	=	fixed(36.55);
calibrate	Galateacrinus	=	fixed(42.3);
calibrate	Gasterocoma	=	fixed(121.7);
calibrate	Glossocrinus	=	fixed(108.65);
calibrate	Graphiocrinus	=	fixed(84.0);
calibrate	Hydreionocrinus	=	fixed(70.0);
calibrate	Indocrinus	=	fixed(18.0);
calibrate	Laudonocrinus=	fixed(36.55);
calibrate	Lecythocrinus	=	fixed(121.7);
calibrate	Lophocrinus	=	fixed(70.0);
calibrate	Manicrinus	=	fixed(156.4);
calibrate	Mastigocrinus	=	fixed(157.5);
calibrate	Oligobrachyocrinus	=	fixed(18.0);
calibrate	Ottawacrinus	=	fixed(180.3);
calibrate	Pachylocrinus	=	fixed(70.0);
calibrate	Paradelocrinus=	fixed(50.40);
calibrate	Pelecocrinus	=	fixed(70.0);
calibrate	Petalocrinus	=	fixed(161.6);
calibrate	Phanocrinus	=	fixed(58.25);
calibrate	Pirasocrinus	= fixed(42.3);
calibrate	Plicodendrocrinus	=	fixed(180.3);
calibrate	Poteriocrinites	=	fixed(70.0);
calibrate	Proctothylacocrinus	= fixed(116.4);
calibrate	Protencrinus	=	fixed(42.3);
calibrate	Rhenocrinus	= fixed(131.65);
calibrate	Rutkowskicrinus	=	fixed(116.4);
calibrate	Scotiacrinus	=	fixed(70.0);
calibrate	Sellardsicrinus=	fixed(42.3);
calibrate	Sostronocrinus	=	fixed(84.0);
calibrate	Scytalocrinus	=	fixed(70.0);
calibrate	Spaniocrinus	=	fixed(11.15);
calibrate	Sphaerocrinus	=	fixed(121.7);
calibrate	Stachyocrinus	=	fixed(11.15);
calibrate	Staphylocrinus=	fixed(58.25);
calibrate	Stellarocrinus	=	fixed(34.18);
calibrate	Streblocrinus	=	fixed(116.4);
calibrate	Sundacrinus	=	fixed(11.15);
```

```
calibrate     Sycocrinites    = fixed(70.0);
calibrate     Texacrinus      =     fixed(42.3);
calibrate     Thalamocrinus=        fixed(150.3);
calibrate     Thenarocrinus =       fixed(161.6);
calibrate     Timorechinus  = fixed(20.45);
calibrate     Ulocrinus       =     fixed(50.40);
calibrate     Zeacrinites     =     fixed(58.25);
delete 51; [delete Ottawacrinus [problematic interpretations of genus morphology]]
[set prior distributions on tree shape and relaxed morphological clock model]
        prset topologypr = constraints(Root, node1, node2, node3, node4, node5, node6,
node7, node8,node9, node10);
        prset treeagepr = fixed(185.0);
        prset nodeagepr = calibrated;
        prset brlenspr = clock : fossilization;
        prset speciationpr = exp(10.0);
        prset extinctionpr = beta(1,1);
        prset fossilizationpr = beta(2,2);
        prset samplestrat = diversity 3: 150.40 0, 90.10 0, 54.4 0; [Ord-Sil/Dev = 150.40,
Dev/Carb = 90.10, Miss/Penn = 54.4]
        prset sampleprob = 0.008; [5/630 = ~0.008]
        prset  clockratepr = normal(0.01, 0.1);
        prset clockvarpr = IGR;
        prset IGRvarpr = exp(10);



[mcmc settings]
        mcmcp ngen= 30000000 samplefreq=3000 printfreq=1000 nruns=2 nchains=4
relburnin=yes burninfrac=0.50 temp = 0.05 savebrlens=yes;
        mcmc;
        sump;
        sumt;

        end;
```

SI References

1. Erwin DH (2007) Disparity: morphological pattern and developmental context. *Paleobiology* 50(1): 57-73.
2. Wagner PJ (2010) Paleontological perspectives on morphologic evolution. *Evolution since Darwin: the first 150 years*, eds. Bell MA et al. (Sinauer, Sunderland), pp 451-478.
3. Foote M (1996) Models of morphological diversification. *Evolutionary Paleobiology*, eds Jablonski D, Erwin DH, Lipps JH (University of Chicago press, Chicago), pp 62-86.
4. Jablonski D (in press) Macroevolutionary theory. *The Theory of Evolution*, eds Scheiner SM, Mindell DP (University of Chicago Press, Chicago).
5. Foote M (1997) The evolution of morphologic diversity. *Annu Rev Ecol Syst* 28(1): 129-152
6. Slater GJ (2015) Not so early bursts and the dynamics nature of morphologic diversification. *Proc Natl Acad Sci USA* 112(12): 4897-4902.
7. Foote M (1996) Models of morphological diversification. *Evolutionary Paleobiology*, eds Jablonski D, Erwin DH, Lipps JH (University of Chicago press, Chicago), pp 62-86.
8. O'Meara BC, Ané C, Sanderson MJ, Wainwright PC, Hansen T (2006) Testing for different rates of continuous trait evolution using likelihood. *Evolution* 60(5): 922-933.
9. Wagner PJ (1995) Testing patterns of evolutionary constraint hypotheses with early Palaeozoic gastropods. *Paleobiology* 21(3): 248-272.
10. Webster GD (2015) Data from "Palaeozoic crinoids, coronates, and hemistreptocrinoids, 1758-2012". Washington State University. crinoids.azurewebsites.net
11. Alroy J (2010) Fair sampling of taxonomic richness and unbiased estimation of origination and extinction rates. *Quantitative Methods in Paleobiology*, eds Alroy J, Hunt G (Paleontological Society Papers 16), pp. 55-80.
12. Foote M (2000) Origination and extinction components of taxonomic diversity: general problems. *Paleobiology* 26(4): 74-102.
13. Bapst DW (2012) Paleotree: an R package for paleontological and phylogenetic analyses of evolution. *Methods Ecol Evol* 3(5): 803-807.
14. Foote M (1999) Morphological diversity in the evolutionary radiation of Palaeozoic and post-Palaeozoic crinoids. *Paleobiology* 25(1): 1-115.
15. Simpson GG (1944) *Major Features of Evolution* (Columbia Univ Press, New York).
16. Ausich WI (1980) A model for niche differentiation in Lower Mississippian crinoid communities. *J Paleontol* 54(2): 273-288.

17. Foote M (1994) Morphology of Ordovician-Devonian crinoids. *Univ Mich Mus Paleontol Contrib* 29(1): 1-39.

18. Wright DF (2015) Fossils, homology, and "phylogenetic paleo-ontogeny": a reassessment of primary posterior plate homologies among fossil and living crinoids with insight from developmental biology. *Paleobiology* 41(4): 570-591.

19. Kammer TW (2008) Paedomorphosis as an adaptive response in pinnulate cladid crinoids from the Burlington Limestone (Mississippian, Osagean) of the Mississippi Valley. *Echinoderm Paleobiology*, eds Ausich WI, Webster GD (University of Indiana Press, Bloomington), pp 177-195.

20. Simpson C (2010) Species selection and driven mechanisms jointly generate a large-scale morphological trend in monobathrid crinoids. *Paleobiology* 36(3), 481-496.

21. Moore RC, Lane NG, Strimple HL (1978) Order Cladida Moore and Laudon, 1943. *Treatise on Invertebrate Paleontology*, *Part T, Echinodermata* 2, ed Moore RC (Geological Society of America and University of Kansas Press, Lawrence), pp T587-T759.

22. Kammer TW, Ausich WI (1992) Advanced cladid crinoids from the Middle Misissippian of the eastcentral United States: primitive grade calyces. *J Paleontol* 66(3): 461-480.

23. Kammer TW, Ausich WI (1993) Advanced cladid crinoids from the Middle Mississippian of the east-central United States: intermediate-grade calyces. *J Paleontol* 67(4): 614-639.

24. Kammer TW, Ausich WI (1994) Advanced cladid crinoids from the Middle Mississippian of the east-central United States: advanced-grade calyces. *J Paleontol* 68(2): 339-35.

25. Kammer TW, Ausich WI (1996) Primitive cladid crinoids from upper Osagean-lower Meramecian (Mississippian) rocks of east-central United States. *J Paleontol* 70(5): 835-866.

26. Wright DF, Ausich WI, Cole SR, Peter ME, Rhenburg EC (2017) Phylogenetic taxonomy and classification of the Crinoidea. *J Paleontol*. 91(4): 829-846.

27. Wright DF (2017) Bayesian estimation of fossil phylogenies and the evolution of early to middle Paleozoic crinoids (Echinodermata). *J Paleontol* 91(4): 799-814.

28. Ausich WI, Kammer TW, Rhenberg EC, Wright DF (2015) Early phylogeny of crinoids within the pelmatozoan clade. *Palaeontol.* 58(6): 937-952.

29. McIntosh GC (2001) Devonian cladid crinoids: families Glossocrinidae Goldring, 1923, and Rutkowskicrinidae new family. *J Paleontol* 75(4): 783-807.
